# The artificial amino acid change in the sialic acid-binding domain of the hemagglutinin neuraminidase of newcastle disease virus increases its specificity to HCT 116 colorectal cancer cells and tumor suppression effect

**DOI:** 10.1186/s12985-023-02276-9

**Published:** 2024-01-04

**Authors:** Bo-Kyoung Jung, Yong Hee An, Sung Hoon Jang, Jin-Ju Jang, Seonhee Kim, Joo Hee Jeon, Jinju Kim, Jason Jungsik Song, Hyun Jang

**Affiliations:** 1 Libentech Co. LTD, Daejeon, Republic of Korea; 2 Graduate School of Medical Science, College of medicine, Yonsei University, Seoul, Republic of Korea; 3 Division of Rheumatology, Department of Internal Medicine, College of Medicine, Yonsei University, Seoul, Korea; 4 Institute for Immunology and Immunological Disease, College of Medicine, Yonsei University, Seoul, Korea

**Keywords:** Directed evolution, Newcastle Disease virus (NDV), Oncolytic virus, Colorectal cancer, Haemagglutinin

## Abstract

**Background:**

Oncolytic viruses are being studied and developed as novel cancer treatments. Using directed evolution technology, structural modification of the viral surface protein increases the specificity of the oncolytic virus for a particular cancer cell. Newcastle disease virus (NDV) does not show specificity for certain types of cancer cells during infection; therefore, it has low cancer cell specificity. Hemagglutinin is an NDV receptor-binding protein on the cell surface that determines host cell tropism. NDV selectivity for specific cancer cells can be increased by artificial amino acid changes in hemagglutinin neuraminidase HN proteins via directed evolution, leading to improved therapeutic effects.

**Methods:**

Sialic acid-binding sites (H domains) of the HN protein mutant library were generated using error-prone PCR. Variants of the H domain protein were screened by enzyme-linked immunosorbent assay using HCT 116 cancer cell surface molecules. The mutant S519G H domain protein showed the highest affinity for the surface protein of HCT 116 cells compared to that of different types of cancer cells. This showed that the S519G mutant H domain protein gene replaced the same part of the original HN protein gene, and S519G mutant recombinant NDV (rNDV) was constructed and recovered. S519G rNDV cancer cell killing effects were tested using the MTT assay with various cancer cell types, and the tumor suppression effect of the S519G mutant rNDV was tested in a xenograft mouse model implanted with cancer cells, including HCT 116 cells.

**Results:**

S519G rNDV showed increased specificity and enhanced killing ability of HCT 116 cells among various cancer cells and a stronger suppressive effect on tumor growth than the original recombinant NDV. Directed evolution using an artificial amino acid change in the NDV HN (S519G mutant) protein increased its specificity and oncolytic effect in colorectal cancer without changing its virulence.

**Conclusion:**

These results provide a new methodology for the use of directed evolution technology for more effective oncolytic virus development.

**Supplementary Information:**

The online version contains supplementary material available at 10.1186/s12985-023-02276-9.

## Background

Colorectal cancer is the third most common cancer type. In 2020, there were 1.9 million new cases of colorectal cancer and 0.9 million deaths worldwide [1]. Its incidence is rapidly increasing in developing countries owing to an increase in meat-based diets. There is an annual 3.3% decrease in colorectal cancer incidence for people over 65 years of age and an annual 1.0% increase for those under 65. Colorectal cancer has the third highest mortality rate among all cancer types. Early stage detection of colorectal cancer leads to a greater than 90% 5-year or longer survival rate of patients, but only 40% of patients are diagnosed with colorectal cancer at an early stage [2].

Since the 1950s, several viruses, including pathogenic ones, have been tested in humans and several species of animals for their cancer cell-killing effect, and applied to human cancer treatment [3, 4]. Several researchers have observed that viruses can impair tumor growth following the cytopathic effects of cancer cells caused by viral infections [5]. Recently, several oncolytic viruses have been developed as virotherapeutics for cancer treatment [6, 7]. Intrinsic oncolytic viruses kill cancer cells without modifying the virus at the molecular level [8]. Adenovirus, adeno-associated virus (AAV), Vaccinia, and herpes simplex virus have been artificially engineered to have an oncolytic effect or an indirect oncolytic effect on cancer cells, such as an immune response enhancement against cancer cells [8, 9]. Nonetheless, the specificity of an intrinsic artificially engineered oncolytic virus to target cancer cells is an important factor in the effectiveness of cancer treatment.

Intravenous injection of an oncolytic virus is an effective treatment for various cancers. To obtain a good therapeutic effect, intravenous-injected oncolytic viruses must have the specificity to target cancer cells. However, most recently developed oncolytic viruses proliferate specifically in cancer cells rather than specifically infecting target cancer cells [10]. If the oncolytic virus has selective cancer cell-proliferation inhibition ability rather than specific cancer cell infection ability, it must be used at high concentrations to obtain the expected efficacy and avoid the two types of dilution effects when the virus is injected through the vein. The first dilution effect occurs in the blood, and a secondary dilution effect occurs due to nonspecific infection of normal cells [11–13]. Using high concentrations of oncolytic viruses can activate an immune response against the virus, reducing its efficacy and causing immune response-related side effects [14, 15]. Another problem raised by the intravenous injection of highly concentrated oncolytic viruses is that repeated inoculations cannot be administered [16, 17]. As a result, currently approved oncolytic virotherapeutics are used for direct tumor injection. The first oncolytic virotherapeutic treatment, Imlygic, was the first approved therapeutic agent for the treatment of melanoma [18]. Since then, more oncolytic viruses have been approved to be used as tumor-directed injectable cancer treatment. However, these are only effective for a limited number of cancer types and cancer progression phases. Therefore, an oncolytic virus is necessary to increase the specificity to target cancer cells and improve efficacy [19].

Directed evolution has been used to adapt the viruses depending on the purpose for which they are used. In 2008, a diverse mutant AAV library was created by DNA shuffling and error-prone PCR, using two different types of AAV cap genes [20, 21]. Specific sialic acid binding affinity increased in the new variant AAV, which has a mutant cap gene, and showed that viruses change their cell tropism induced by an artificial selective mutation in the virus receptor. However, gene-based directed evolution has not been used to alter the specificity of oncolytic viruses [22, 23]. Several attempts are currently being made to enhance the affinity of oncolytic viruses to specific cancer cells through successive passage cultures using the virus and cancer cells of interest. In 2019, a subsequent passage culture method was used to increase the specificity of the oncolytic virus to a specific cancer cell. However, subsequent passage culture of the virus is not an established method for generating an oncolytic virus with a specific ability to infect cancer cells [24] because viral receptor changes during successive passage culture are difficult to control, especially on the mutation site, which is a critical target amino acid on the viral receptor. Moreover, mutations must only affect cancer cell specificity and not cause other changes, such as pathogenicity. NDV is an oncolytic virus, meaning that it can selectively infect and replicate in tumor cells while sparing healthy cells. This selectivity is crucial for their potential use in cancer therapy. NDV primarily enters host cells through interactions between its surface glycoproteins, particularly hemagglutinin-neuraminidase (HN), and host cell surface receptors. The specificity of these interactions determines the number of cells into which the virus can enter. NDV may use different receptors on tumor cells than on normal cells, leading to selective attachment. Tumor cells often exhibit different patterns of receptor expression compared to normal cells. These differences may include overexpression of certain receptors or the presence of unique receptors that are not abundant in healthy cells. The NDV may have a preference for these receptors, allowing it to selectively infect tumor cells. Achieving precise tumor targeting while sparing healthy tissues is a significant challenge. Although NDV can exhibit tumor selectivity, it may still infect normal cells to some extent. Enhancing the specificity of cancer cells is crucial for minimizing the potential side effects. NDV also have weak specificity for target cancer cells and show strong multiplication ability [25]. NDV is an intrinsic oncolytic virus that exhibits good efficacy. However, several studies have used wild-type or attenuated vaccine strains; therefore, NDV also exhibits the same dilution-effect challenges when administered intravenously [26–28]. To date, the directed evolution method has not been used to increase the specificity of NDV for targeting cancer cells. Furthermore, increased virus specificity to target cells is important for developing virotherapeutics for the treatment of cancer and genetic disorders [29, 30]. Fortunately, NDV does not induce immune responses in mammals but requires cancer cell specificity because although NDV does not propagate it can infect normal mammalian cells. Moreover, NDV has no specificity for cancer cells, so wild-type and attenuated NDV exert intrinsic oncolytic effects on all cells. Some NDV-based cancer treatment studies have been performed in the clinical phase, but are still not commercialized [31–34].

NDV is divided into three categories according to its pathogenicity, and the intrinsic oncolytic activity of NDV depends on its virulence [35]. Propagation and reinfection of neighboring cancer cells are more active in high-virulence NDV than in low-virulence NDV [36]. Several studies have found that NDV virulence is closely related to oncolytic activity, with high-virulence NDV typically exhibiting a higher oncolytic activity. HN and F proteins are essential for NDV pathogenicity. However, it is still unclear which amino acids in the HN and F proteins are related to the pathogenicity of NDV [37, 38] and how they increase oncolytic activity at the molecular level [39]. NDV infection is initiated by binding of the HN protein to sialic acid on the cell surface. Molecular-level analysis of the HN protein and its binding mechanism with sialic acid provides information on how the HN protein specifically binds to sialic acid and has multifunctionality. Moreover, it was found that specific amino acids in the globular head region of the HN participated in sialic acid binding. The binding of sialic acid to HN causes a conformational change, creating new sialic acid-binding sites at the dimeric interface. Sialic acid binding induces neuraminidase activity and tetramer formation. Subsequent interaction between HN and the fusion protein promotes a fusogenic state, and the RNA genome of the NDV enters the cytoplasm of the infected cell [40–42]. The types of cells infected with the NDV are relatively diverse, and it is assumed that the HN protein is not specific to the type of sialic acid [43]. NDV must meet two requirements as a good virotherapeutic for cancer treatment. First, NDV must infect specific cancer cells and increase its oncolytic activity without causing side effects. The HN protein is an excellent candidate for directed evolution, which involves modification of a receptor-binding motif with cancer cell specificity.

In this study, we created a mutant HN protein harboring recombinant NDV with increased specificity for specific cancer cells by artificially mutating the sialic acid-binding site of the HN protein using error-prone PCR. We propose library construction and enzyme-linked immunosorbent assay (ELISA) screening methods for mutants with increased cancer cell specificity in mutant libraries. Our results showed that NDV with the mutant HN protein with increased binding ability to cancer cell surface proteins enhances the cancer cell-killing effect and improves the tumor suppression effect.

## Methods

### Error-prone polymerase chain reaction of the sialic acid binding motifs (H domain) gene

Amino acid sequences 123 to 571 (UniProt Acc. No. P13850) of hemagglutinin-neuraminidase, the sialic acid-binding domain of the Newcastle disease virus gene, was optimized according to codon use in *E. coli*. The nucleotide production company synthesized that sequence with two restriction enzyme sequences (BamHI sequence at 5 at 5oSbfI sequence at 3at 3Bioneer, Korea). The synthesized gene was subjected to error-prone PCR to construct a mutant gene library. Error-prone PCR (EP-PCR) conditions were set according to the EP-PCR kit protocol (GeneMorph II Random mutagenesis kit, Agilent). The mutation rate was 0 ~ 4.5 mutations/kb, and the EP-PCR primer set was H domain EP-PCR F 5`- GGATCCTGTGGTGCGCCAATTCATG − 3` and H domain EP-PCR R 5`- CTGCAGGACTCCATCATCCTTCAGAAT − 3.’

### Expression of the mutant H domain library

ELISA was performed against the surface protein of HCT 116 cells to determine the increased affinity of the mutant H domain library. The whole monolayer of HCT 116 cell was harvested and centrifuged to collect the cell contents. HCT 116 cell surface proteins were isolated using a cell-surface protein isolation kit (ab65400 Plasma Membrane Protein Extraction Kit; Abcam. Co.). This process was performed according to the manufacturer’s protocol, and the concentration of isolated cell surface proteins was measured using the Lowry method. The final concentration of the cell surface protein is adjusted to 50 µg/ml with ELISA coating buffer (bicarbonate/carbonate (Na_2_CO_3_/NaHCO_3_) coating buffer, 100 mM, pH 9.6). Then, 100 µl of the cell surface protein was added to 96-well plates and incubated at 4 ℃ overnight. The next day, the cell surface protein solution was discarded, and 200 µl of the blocking solution (phosphate-buffered saline, pH 7.4, 1% BSA, 0.05% Tween 20) was added and then incubated at room temperature for 1 h. The blocking solution was discarded and 100 µl of the mutant H domain-containing *E. coli* lysate solution (prepared experimental method No. 2), add to 96-well plates, and incubate at room temperature for 1 h. Discard the solution and wash the plate three times with washing buffer (phosphate-buffered saline, pH 7.4, 0.05% Tween 20). One hundred microliters of (horseradish peroxidase (HRP)-conjugated biotin-containing solution were added to 96-well plates and incubated at room temperature for 1 h. The HRP-conjugated biotin solution was removed from the plate and washed thrice with washing buffer. Fifty microliters of substrate (TMB. 3,3’,5,5’-Tetramethylbenzidine, Invitrogen) was added to the 96-well plates, and 50 µl of the stop solution (2.0 N, H_2_SO_4_) was added to the 96-well plates after a 10-min color development step. Absorption at 450 nm in each well was read using an ELISA plate reader (iMark™ Microplate Absorbance Reader, Bio-Rad). We selected several high optical density (OD) absorbance-containing H domain mutants from colonies in the transformed *E. coli*. Then, to identify the mutant gene sequences the mutant with the highest absorption was determined and traced.

### Colorectal cancer cell specific affinity increased mutant H domain screening

ELISA was performed against the surface protein of HCT 116 cells to determine the increased affinity of the mutant H domain library. The whole monolayer of HCT 116 cell was harvested and centrifuged to collect the cell contents. HCT 116 cell surface proteins were isolated using a cell-surface protein isolation kit (ab65400 Plasma Membrane Protein Extraction Kit; Abcam. Co.). This process was performed according to the manufacturer’s protocol, and the concentration of isolated cell surface proteins was measured using the Lowry method. The final concentration of the cell surface protein is adjusted to 50 µg/ml with ELISA coating buffer (bicarbonate/carbonate (Na_2_CO_3_/NaHCO_3_) coating buffer, 100 mM, pH 9.6). Then, 100 µl of the cell surface protein was added to 96-well plates and incubated at 4 ℃ overnight. The next day, the cell surface protein solution was discarded, and 200 µl of the blocking solution (phosphate-buffered saline, pH 7.4, 1% BSA, 0.05% Tween 20) was added and then incubated at room temperature for 1 h. The blocking solution was discarded and 100 µl of the mutant H domain-containing *E. coli* lysate solution (prepared experimental method No. 2), add to 96-well plates, and incubate at room temperature for 1 h. Discard the solution and wash the plate three times with washing buffer (phosphate-buffered saline, pH 7.4, 0.05% Tween 20). One hundred microliters of (horseradish peroxidase (HRP)-conjugated biotin-containing solution were added to 96-well plates and incubated at room temperature for 1 h. The HRP-conjugated biotin solution was removed from the plate and washed thrice with washing buffer. Fifty microliters of substrate (TMB. 3,3’,5,5’-Tetramethylbenzidine, Invitrogen) was added to the 96-well plates, and 50 µl of the stop solution (2.0 N, H_2_SO_4_) was added to the 96-well plates after a 10-min color development step. Absorption at 450 nm in each well was read using an ELISA plate reader (iMark™ Microplate Absorbance Reader, Bio-Rad). We selected several high optical density (OD) absorbance-containing H domain mutants from colonies in the transformed *E. coli*. Then, to identify the mutant gene sequences the mutant with the highest absorption was determined and traced.

### Confirmation of the S519G mutant H domain protein specific binding to HCT 116 colorectal cancer cell

S519G mutant H domain gene containing pRSETA plasmid vector was transformed to *E. coli* (BL 21) in 100 ml of LB broth (100 µg/ml ampicillin) and cultured while on a shaking incubator (37 ℃, 200 rpm) until the optical density at 600 nm reached 0.4 to 0.8. After the optical density reached 0.4 to 0.8, 100 µl of the 0.1 M IPTG was added and left to incubate for another 3 or 4 h at 27 ℃, 100 rpm. The culturing step was completed with the collection of the cell precipitate from the E. coli cell mixture centrifugation (4 ℃, 6,000 g, 10 min). The collected E. coli cells were resuspended in 20 ml of PBS containing a protease inhibitor (Protease Inhibitor Cocktail, Sigma-Aldrich) and lysed (40 W, 5 min, 10 s on– 10 s off) using a sonicator (Q125 sonicator, Qsonica). The lysed solution was centrifuged and the supernatant was separated from the precipitate. Ammonium sulfate was added to the supernatant, and the pellet was separated by centrifugation (4 ℃, 8,000 g, 10 min). The pellet was re-suspended in 50 mM HEPES buffer (pH 6.5) and dialyzed with 20 mM HEPES buffer (pH 6.5). The S519G mutant H domain protein expressed with avidin was purified using cation-exchange chromatography (GE Healthcare, HiTrap SP FF, 5 ml). After sample loading, the S519G mutant H domain protein was eluted with 1.0 M NaCl containing HEPES buffer (pH 6.5), and the fractions (1.0 ml) were monitored by measuring UV absorption at 280 nm. S519G mutant H domain-containing fractions were mixed and polished by size exclusion chromatography (GE healthcare Superdex™ 200 Increase column), and a cancer cell-specific binding test was used.

ELISA was performed using various cancer cell surface proteins to confirm the specific binding affinity of the S519G mutant H domain protein to HCT 116 colorectal cancer cells. The cancer cell lines used in the experiment were HCT 116, HT-29, SW620, A549, and T98G, and the normal cell lines were CCD-18Co and MRC-5. Cancer cell surface proteins were extracted using a plasma protein extraction kit (Abcam), and protein concentration was measured using the Lowry protein assay. Cancer cell surface protein concentration was adjusted to 50 µg/ml with a coating buffer (bicarbonate/carbonate (Na_2_CO_3_/NaHCO_3_) coating buffer, 100 mM, pH 9.6). 100 µl of cancer cell surface protein solution was dispensed on a 96-well ELISA plate, and plate coating was done at 4 ℃ overnight. The coating solution was discarded and blocking, washing, S519G mutant H domain binding, HRP-conjugated biotin binding, and color development were performed following the general ELISA method.

### The S519G mutant H domain protein specific binding test to live HCT 116 colorectal cancer cells using enhanced green fluorescent protein (EGFP) linked S519G mutant H domain protein

To confirm the specific binding of the S519G mutant H domain protein to the colorectal cancer cell HCT 116, a green fluorescent protein-linked S519G mutant H domain fusion protein was constructed. The enhanced EGFP gene was codon-optimized following codon use in the synthesized *E. coli* (Macrogen, Republic of Korea). The S519G mutant H domain gene was linked to the EGFP gene using a linker peptide gene and transformed into *E. coli* for protein expression. S519G mutant-EGFP gene was inserted into the plasmid vector pET15b and transformed into *E. coli*. The *E. coli* was spread over LB agar media containing 100 µg/ml of ampicillin and incubated at 37 ℃ overnight. The colony-forming *E. coli* was selected and inoculated into LB broth containing 100 µg/ml ampicillin. Once optical density reached 0.4 at 600 nm, a final concentration of 0.1 M IPTG was added to the culture to promote protein expression. All other protein expression and purification processes were performed following method 4. S519G mutant H domain-avidin protein preparation through ion-exchange chromatography. Specific binding test of the S519G mutant H domain-EGFP protein to HCT 116 was done with cancer cells (T98G and A549) and normal cells (CCD-18Co). Test cells were cultured in a 96-well cell culture plate for 24 h before testing by following each cell subculture method. Monolayer-forming cells on the plate were removed from the media, washed with PBS, and fixed with fixation buffer (methanol and acetone 1:1) at -20 ℃ for 10 min. Then, 5 µg/50 µl of the purified S519G mutant H domain-EGFP protein was attached at 37 ℃ for 1 h to the cells and washed twice with PBS. The cell plates were observed using a DMi8M fluorescence microscope (Leica, Germany) equipped with a DFC 3000 G digital camera.

### Rescue of the recombinant NDV containing H domain replaced by the S519G mutant H domain

The NDV VG/GA cDNA sequence replaced thymine and cytosine with guanine at 1,555 and 1,556. For base sequence changes, the site-directed mutagenesis cloning method was used with the recombinant NDV (rNDV) vector [44, 45]. In this study, the HN mutation primers forward 5`-CTAGAGCAGAGGAAAAGATTAC-3` and reverse 5`-AGATCGCAGTCGGTATGCCTACAA-3` were used. Sequencing confirmed that the nucleotide swapping happened appropriately. The constructed vector was named the S519G mutant rNDV. HEp2 cells were cultured in a 6-well cell culture plate (seeding density: 5 × 10^5.0^ cells/well). The monolayer-forming HEp2 cells were inoculated with modified Vaccinia virus (MVA/T7) at 0.1 multiplicity of infection (MOI) and incubated at 37 ℃ (5% CO_2_) for 2 h. Post inoculation, the cells were washed with PBS (pH 7.4) and added 2.0 ml of minimum essential media (Gibco) containing 1% fetal bovine serum (Gibco) and 1% penicillin solution (Gibco, 10,000 unit/ml of penicillin and 10,000 µg/ml of streptomycin) to the well. Immediately after, 250 µl of premixed Lipofectamine 3000 (Invitrogen), P3000 reagent (Invitrogen), S519G mutant rNDV viral vector (5 µg), and three helper plasmids (NP 2.5 µg, *P* 1.5 µg, and L 0.5 µg, respectively) were added per well and the plate was incubated on a 37 ℃ incubator (5% CO_2_). The supernatant was harvested 96 h post-transfection and 200 µl of the supernatant was used to inoculate the allantoic cavity of 8–10 days-old embryonated SPF eggs. After 96 h of inoculation, the allantoic fluid was harvested, and repeated inoculations were administered to 8–10 days-old embryonated eggs. The harvested allantoic fluid was tested for recovered recombinant NDV, and MVA/T7 was removed using RT-PCR (Bioneer) and PCR (KOD One™, TOYOBO, Japan) according to the manufacturer’s instructions. The primers used in this study are listed in Supplementary Data 1.

### Purification of the Recombinant virus

The S519G mutant rNDV and rNDV viruses were cultured on Vero cells (KCLB 10,081). Vero cells were prepared in a 1 × 10^7.0^ cells/175 flask. More than 80% of the monolayer-forming Vero cells were washed with PBS and inoculated with 0.5 MOI. After inoculation, the infection step included gentle shaking at 15-minute intervals for 1 h. After infection, the virus culture medium was removed, cells were washed once with PBS, and fresh medium was added. After two days, the supernatant containing the virus was harvested. Harvesting the virus culture was centrifuged at 3,000 g, 4 ℃ for 10 min and separating the supernatant. Virus in the supernatant was purified by size exclusion chromatography (GE Healthcare, HiScreen™ Capto™ Core 400). The column was loaded with 25 ml of supernatant and eluted with 20 mM Tris-HCl (pH 7.5) buffer containing 150 mM NaCl at a flow rate of 1.6 ml/min. Virus-containing fractions were recovered by UV absorption at 260 and 280 nm. Virus-containing fractions were ultracentrifuged at 80,000 g, 4 ℃ for 3 h, and re-suspended in TNE buffer (10 mM Tris-HCl, 20 mM NaCl, and 1 mM EDTA). The concentrated virus was polished by ultracentrifugation at 100,000 g at 4 ℃ for 2 h using a 10 to 50% sucrose linear gradient method. After centrifugation, the white band of the centrifuge tube was separated and dialyzed against PBS (pH 7.4) to remove sucrose.

### Median death time (MDT) test

Median death time (MDT) was measured according to the previously published method [46]. The exact concentration of recombinant NDV (rNDV) was used as a control for the MDT measurement test. The titers of the two viruses, rNDV and S519G mutant rNDV, were adjusted to the same concentration of 10^6.3^ TCID_50_/ml and inoculated with no dilution and 1/10 and 1/100 dilutions. Then, 0.2 ml of virus was inoculated in the allantoic cavity of 9 to 11-day-old SPF embryonated eggs (six eggs for one concentration unit of each virus) and cultured in an egg incubator (Ari 50, evergreen farm) at 37 ℃ for 7 d. MDT was determined by the average death time of the embryonated egg, showing the blood vessels of the egg; however, the inoculation made on dead eggs within 24 h were not counted. The HA titer was used to immediately measure the allantoic fluid from dead eggs. HA titers were measured according to a previously published method [47]. The allantoic fluid (25 µl) was serially two-fold diluted in a U-shaped 96-well plate with an equal amount of PBS and 25 µl of the 1% chicken red blood cells in PBS. At least 75% of the area of full hemagglutination observed in the wells was counted for HA titer determination.

### Intracerebral pathogenicity index (ICPI) test

The intracerebral pathogenicity index (ICPI) of the S519G mutant rNDV was measured using the OIE Terrestrial Manual 2021. 0.05 ml of the S519G mutant rNDV virus (10^6.3^ TCID_50_/ml, ≥HA titer 26) and rNDV virus, the same concentration of the S519G mutant rNDV inoculated into the brain (intracerebral injection) of 1-day-old SPF chickens (10 chicks for each virus). The chicks were observed for clinical symptoms and death every 24 h for 8 d post-inoculations. A healthy chicken was assigned a score of 0, a sick chicken was assigned a score of 1, and a dead chicken was assigned a score of 2. The ICPI of the virus was determined based on the mean scores of chicks in the same group.

### Analysis of viral growth

Viral growth was detected in Vero cells. For the experiment, cells were seeded in a 6-well plate and infected with a viral load of 0.5 MOI of rNDV or S519G mutant rNDV viruses. Supernatants were harvested at 24, 48, 72, and 96-h post-infection. Subsequently, the viral titer was determined at 50% tissue culture infective dose per milliliter using Vero cells as the indicator cells.

### Cell viability test

The cytopathic effect (CPE) was identified, and the MTT assay was performed. CPE was identified in HCT 116, HT-29, CCD18-Co, A549, and T98G cell lines. The MTT assay was performed in HCT 116, HT-29, CCD-18Co, A549, and T98G according to a conventional method and described the detailed experimental procedure. 1 × 10^4^ cells/well of HCT 116, HT-29, CCD18-Co, A549, and T98G were plated in a 96-well plate for 24 h and cultured in a 37 ℃ incubator (5% CO_2_), and rNDV and S519G mutant rNDV viruses were infected at 0.01, 0.1, 1, and 2.5 MOIs. To ensure reliability of the results, four wells were prepared under the same conditions. After infection, the cells were cultured in a 37 ℃ incubator (5% CO_2_), and 48 and 96 h after infection, 20 µl of MTT solution (CellTiter 96® AQueous One solution Cell Proliferation Assay, Bio-Rad, USA) was added to each well. The cells were incubated for 1 h in an incubator (5% CO_2_). Cell death was determined by measuring the absorbance at 490 nm using an iMark Microplate Reader (Bio-Rad). The relative death rate (%) in the negative control group was also determined.

### Animals

All animal experiments conducted in this study were approved by the Animal Care and Use Committee of Libentech Co., Ltd. (LBT-IACUC-AE-2021-02). Five-week-old female BALB/c nu-/nu- mice were purchased from Orient Bio (Seoul, Republic of Korea). Throughout the study, the mice were housed in controlled conditions with an ambient temperature of 22 ± 1 ℃ and a light/dark cycle of 12/12 h. They were provided free access to sterilized food and water and were monitored regularly for signs of distress or adverse effects. A body-weight reduction of 20% or more was considered a human endpoint, at which the mice were sacrificed to minimize potential suffering and ensure animal welfare during the experimental period.

### Animal studies

To construct the xenograft mouse model, 100 µl of HCT 116, HT-29, and A549 cancer cell cultures (1×10 ^7.0^ cells/ml in RPMI media) were mixed with an equal volume of Matrigel (Corning) and implanted into the mice’s left hip. Twelve SPF female BALB/c nude mice were randomly divided into groups of four mice per group. Each experimental group was further divided into groups in which the rNDV virus, S519G mutant rNDV virus, and PBS were intravenously injected into the tail vein. The concentration of the inoculated virus was 10^7.0^ TCID_50_/dose. The tumor growth rate was compared between the treatment and control groups after inoculations. Virus inoculation was performed three times at 2-d intervals from when the cancer tissue size reached an average of 50 200 mm^3^ on the 5th day after cell inoculation. Seventeen days after virus inoculation, changes in the cancer tissues were observed every two days. The last change in cancer tissue was observed on day 17 after virus inoculation. The size of the cancerous tissue was calculated using the formula 1/2 × (smallest diameter)^2^ × (largest diameter).

### Immunohistochemical (IHC) assay

The tumor tissues were fixed using a 10% (w/v) formalin solution and processed for paraffin embedding. Tumor blocks were dehydrated using ethanol and xylene. The HN antigens in tissues were localized for immunohistochemical (IHC) staining. The 4-µm-thick sections were prepared and incubated overnight at 4 ℃ with primary antibodies against NDV HN (1:1,500, BS-4529R, Bioss, USA). The color was observed using 3,3′-diaminobenzidine (DAB). All images were detected using Image J V.1.8.0.

### Statistical analysis

All data are presented as the mean ± the standard error of the mean (SEM). Statistical comparisons were performed using Student’s t-test or one-way ANOVA using SPSS (Version 23, SPSS, Inc., Chicago, IL, USA). Statistical significance was set at *P* < 0.05.

## Results

### H domain mutant library construction and mutant protein expression

**Fig. 1:**
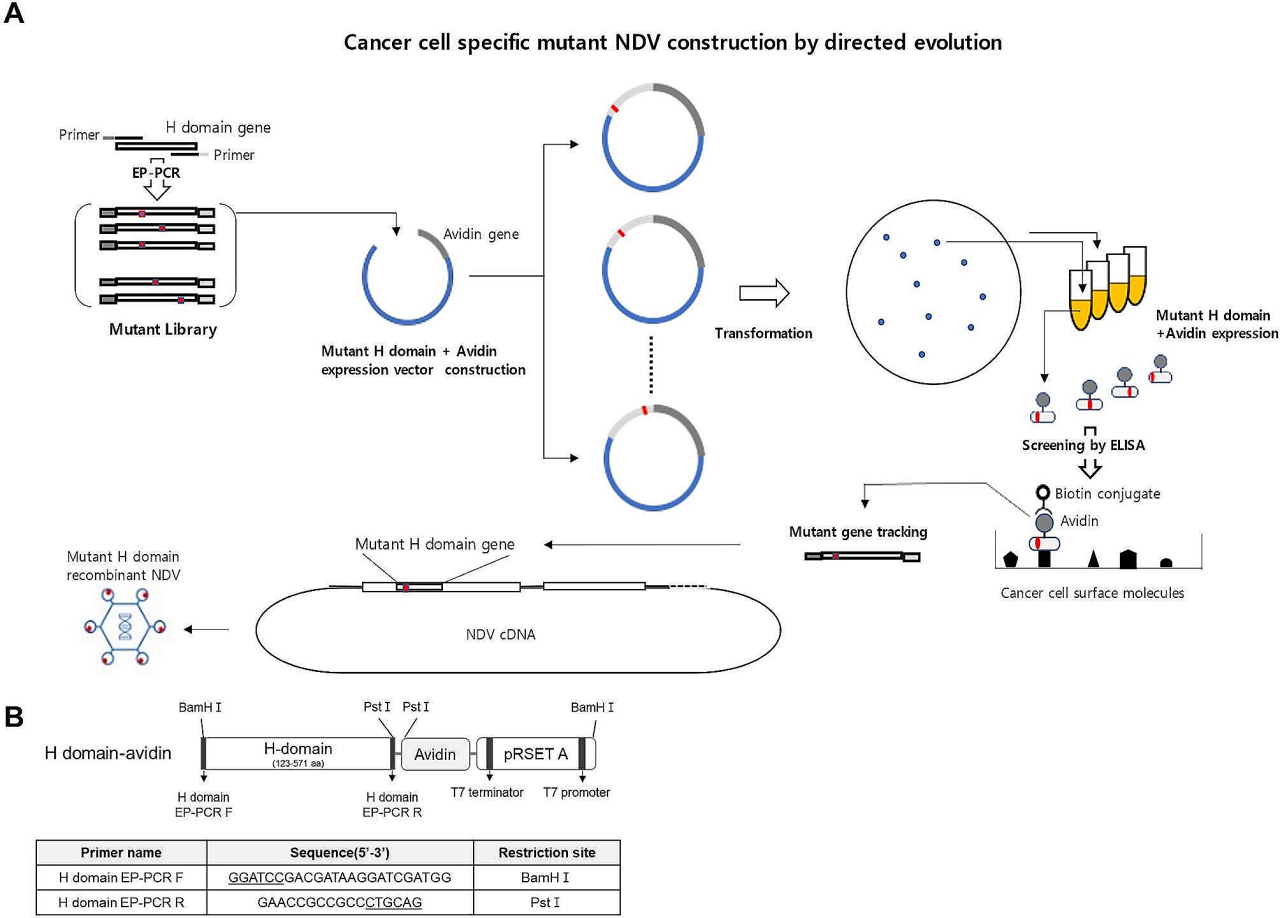
Directed evolution. (**A**) Schematic diagram of directed evolution technology to construct an H domain protein with the improved binding affinity to cancer cells. (**B**) Schematic diagram of the H-domain-avidin protein. H-domain, the globular head domain

H domain gene mutant libraries were constructed using error-prone PCR (EP-PCR) and expressed in *E. coli* expression systems. Based on ELISA results, cancer cell surface proteins were used to identify a cancer-specific H domain with improved binding affinity (Fig. 1A). The EP-PCR mutation rate is 0 ~ 4.5 mutations/kb in the H domain gene by random sample sequencing (data not shown). The mutated H domain genes were inserted into the pRSET-A plasmid vector for *E. coli* expression. The mutant H domain was expressed with avidin and linked using a peptide linker (Fig. 1B). The pRSET-A vector-transformed *E. coli* were spread over the LB media plate, and approximately 200–300 colonies were formed per plate. Each colony was picked, inoculated on LB medium, and marked to trace the mutant H domain gene. Mutant H domain-avidin proteins are expressed as a mixture of soluble and insoluble proteins.

In many cases, H domain avidin proteins are expressed as insoluble proteins. In this study, soluble H domain-avidin proteins were found in the supernatants of centrifuged samples used for the ELISA screening. Several million mutants are typically produced by EP-PCR. However, the number of mutants that can be screened using the *E. coli* expression system is less than 0.1%. We screened approximately 10,000 mutant colonies in this study, which represented a relatively small part of the entire mutant library; however, the number of feasible screenings was limited by current laboratory technology.

### Mutant H domain screening against the surface proteins of HCT 116 colorectal cancer cells using ELISA

The mutant library was screened against HCT 116 cell surface proteins using ELISA. Soluble mutant H domain-avidin proteins isolated from *E. coli* were used for affinity-increased mutant screening against the surface proteins of HCT 116 cells. Mutant H domain-avidin proteins isolated from each colony were adjusted to the same protein concentration (50 µg/mL) and added to the wells coated with the HCT 116 cell surface proteins. Using ELISA, relatively high absorbance was observed in marked, traced, and sequenced colonies. The colony containing the mutant H domain gene was sequenced, and several mutants showed relatively high absorbance. Mutant H domain gene sequence analysis showed that the locations of mutations were very diverse and no specific pattern was observed (data not shown). Sequence analysis of the highest absorbance of the mutant H gene showed that the mutation occurred at amino acid position 519. The absorbance of the S519G mutant H domain was 1.28 at 450 nm (Supplementary Data 2).

### Specific binding tests of S519G mutant H domain against several cancer cell surface proteins using ELISA

**Fig. 2:**
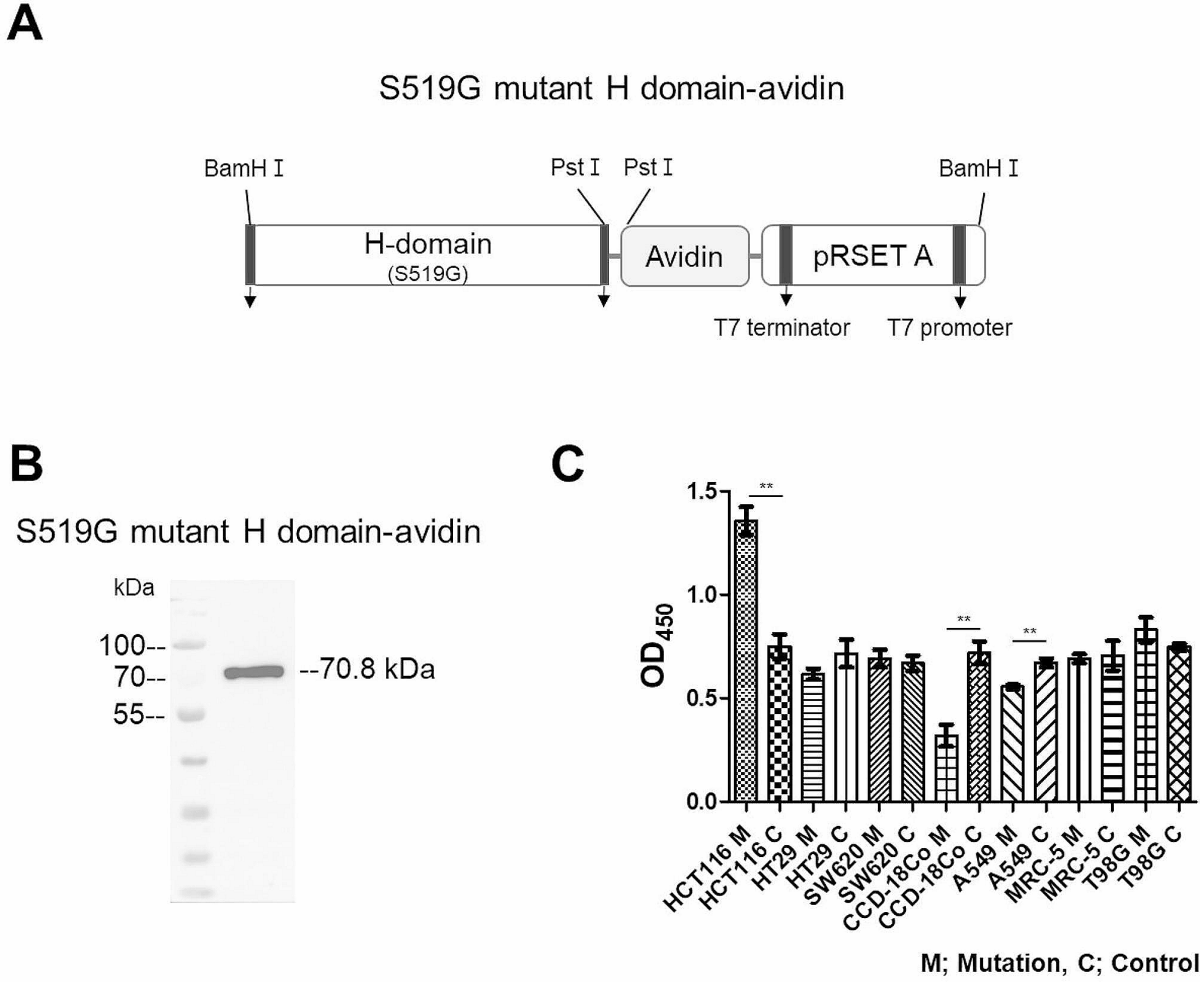
Binding of the S519G mutant H-domain-avidin protein to the cancer cell surface. (**A**) Schematic diagram of the S519G mutant H-domain-avidin protein. The amino acid at positions 519 of the H domain was mutated from serine to glycine. The S519G mutant H-domain-avidin protein were expressed and purified. (**B**) The protein was purified with ion exchange chromatography and analyzed by western blot. (**C**) The binding ability of the H-domain mutation-avidin protein. The protein was identified in colorectal cancer cells (HCT 116, SW620, HT29), colorectal normal cells (CCD18-Co), other cancer cells (A549; lung cancer, T98G; glioblastoma) and normal cells (MRC-5; lung normal cells). (n = 4, bar: SEM, *p < 0.05, **p < 0.01, ***p < 0.001 determined by the Student’s t-test). H domain, the globular head domain; M, HN mutation; C, control

To confirm the increased affinity of the S519G mutant H domain protein for HCT 116 cell surface proteins, ELISA was conducted with various cancer cells and normal cell surface proteins. The purified soluble form of the S519G H domain-avidin protein (Fig. 2A) was identified using western blot analysis (Fig. 2B) and was further used for ELISA. As shown in Fig. 2C, the S519G H domain-avidin protein showed the highest affinity for HCT 116 cell surface proteins compared with that of the other colon cancer cells (HT-29 and SW620), normal colon cells (CCD-18Co), various other cancer cells (A549; lung cancer cells, MRC-5; normal lung cells, T98G; glioblastoma cells), and normal cells (MRC-5; normal lung cells). The highest OD value was observed for HCT 116 cell surface proteins. The lowest absorbance was observed in the A549 (lung cancer cells) and MRC-5 (normal lung cells). ELISA showed that the S519G mutant H domain-avidin proteins had a specific binding affinity to HCT 116 cells compared to the original H domain-avidin protein. Absorbance at 450 nm of the S519G mutant H domain-avidin protein was 1.39 against the HCT 116 cell, but the same colon tissue-originated normal cell, CCD-18Co, showed an absorbance of 0.32 at 450 nm. There was no significant difference in the absorbance of the original H domain-avidin protein between colorectal cancer cells and normal cells, with a 0.75 and 0.72 absorbance for the HCT 116 colorectal cancer cell and CCD-18Co normal colon cell. Except for HCT 116 colorectal cancer cells, the absorbance of the S519G mutant H domain-avidin protein measured via ELISA was observed to be between 0.56 and 0.83 when using the surface proteins of several types of cancer and normal cells. In particular, A549 lung cancer cells showed different results from those of colon cancer cells. The absorbance of the original H domain-avidin protein was 0.67, but the absorbance of the S519G mutant H domain-avidin protein had a low value of 0.49. In HCT 116 and T98G cells, the S519G mutant H domain showed a higher OD value than the normal H domain. In A549 and CCD-18Co cells, the normal H domain showed a higher OD value than the S519G mutant H domain. However, in T98G cells, the OD value of the S519G mutant H domain was 0.08 higher than that of the normal H domain. Based on the ELISA results, serine replaced by glycine in the H domain influences the binding affinity of the S519G H domain to specific cancer cells, such as HCT 116 cancer cells.

### S519G mutant H domain specific bonding against HCT 116 cell surface using fluorescence microscopy

**Fig. 3:**
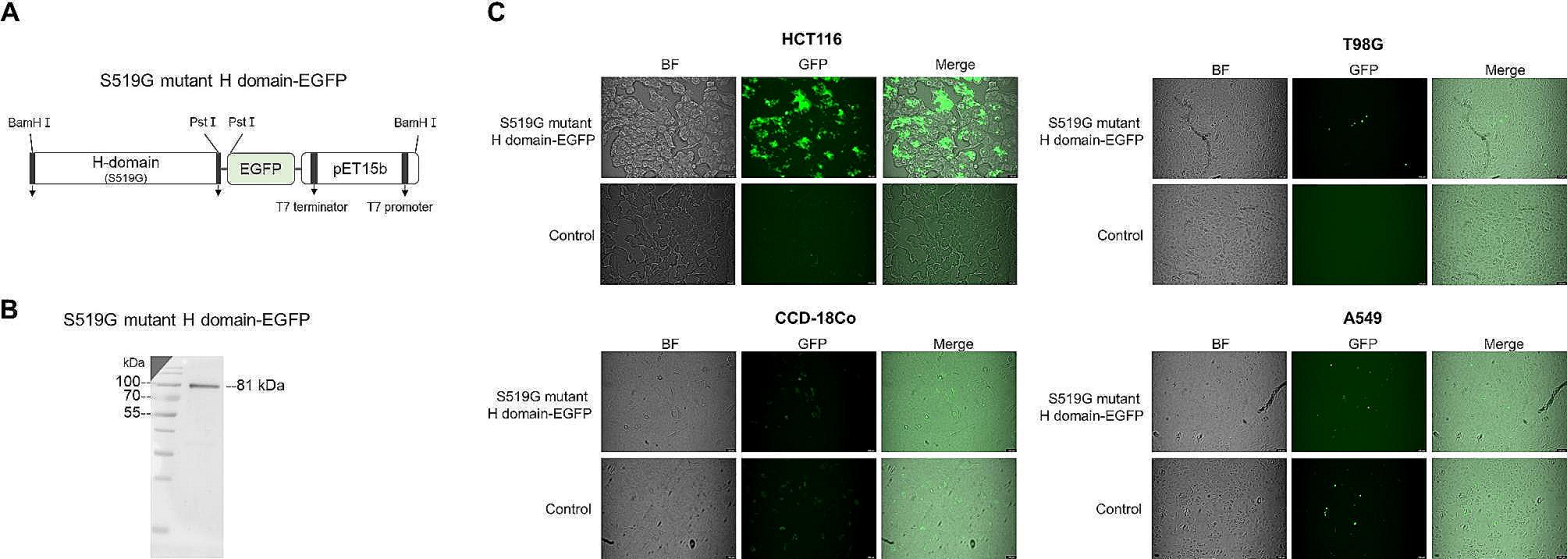
Binding of the S519G mutant H-domain-EGFP protein to the cancer cell surface. (**A**) Schematic diagram of the S519G mutant H-domain-EGFP protein. The amino acid at positions 519 of the H domain were mutated from serine to glycine. The S519G mutant H-domain-EGFP protein was expressed and purified. (**B**) The purified S519G mutant H-domain-EGFP protein was analyzed by western blot. (**C**) The binding of the S519G mutant H-domain-EGFP protein on various cancer cells. The surface binding of the S519G mutant H-domain-EGFP protein on various cancer cells (HCT 116: colorectal cancer, T98G; glioblastoma, A549; lung cancer) and colorectal normal cells (CCD-18Co) of a S519G mutant H-domain-EGFP protein for various cancer cells using a fluorescence microscope. H domain, the globular head domain. EGFP; enhanced green fluorescent protein

To confirm that the S519G mutant H domain binds specifically to HCT 116 cells, the S519G mutant H domain protein linked with the EGFP fusion protein was expressed in *E. coli* for the binding test to the living cell surface (Fig. 3A). The S519G mutant H domain green fluorescent protein (GFP) was expressed and purified (Fig. 3B).

Based on the ELISA results, normal colon cells (CCD-18Co) and two other types of cancer cells (A549 and T98G) were chosen for fluorescence intensity comparison test with HCT 116 cells (Fig. 3C). The 519G mutant H domain-GFP attached to HCT 116 cells showed strong fluorescence. However, fluorescence was either slightly or not at all observed or was only slightly observed in normal colon cells (CCD-18Co) and other cancer cells (A549 and T98G). The fluorescence observation results were similar to those of the ELISA. However, in the case of T98G cells, unlike the ELISA results, fluorescence was not detected for H domain-GFP when observed with a fluorescence microscope, and weak (slight) fluorescence was observed for the S519G mutant H domain-GFP.

### S519G mutant rNDV virus recovery and growth kinetics

**Fig. 4:**
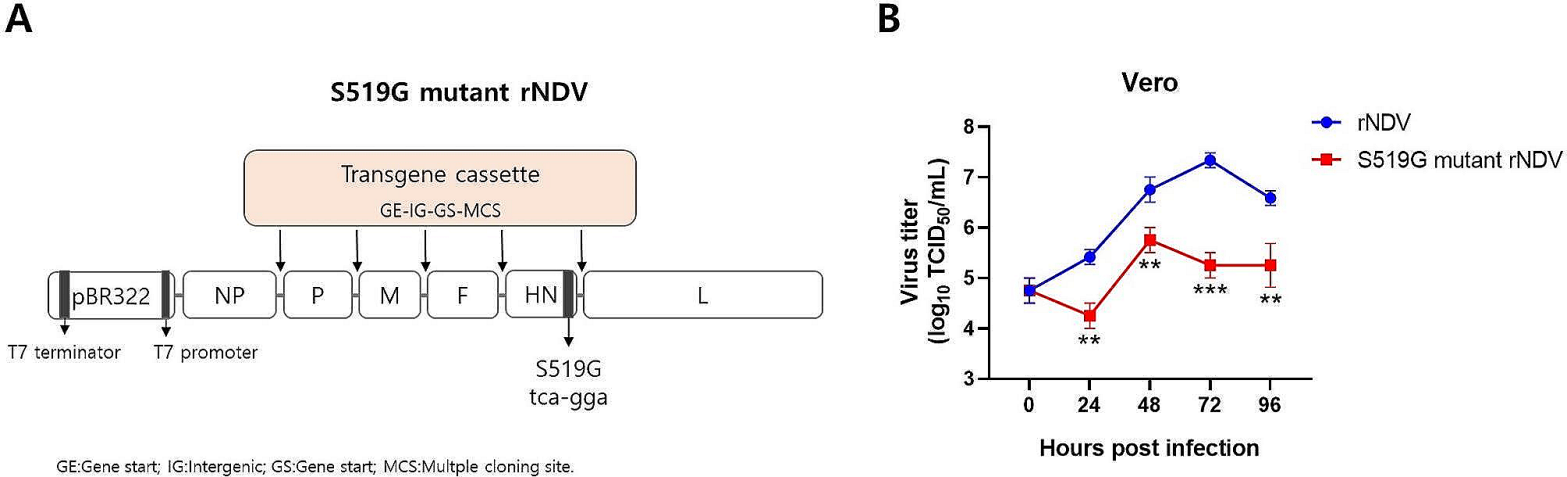
The construction of the S519G mutant rNDV virus. (**A**) Schematic diagram of the S519G mutant rNDV plasmid. The transgene cassette is composed of a GE-IG-GS sequence and a multi cloning site (MCS) in front of the N-terminus of the foreign gene insertion site, and can be constructed by inserting the transgene cassette between the NP and P genes, the P and M genes, the M and F genes, the F and HN genes, and the HN and L genes. (**B**) Growth kinetics of rNDV and S519G mutant rNDV viruses in Vero cells. The cells were respectively infected with rNDV and S519G mutant rNDV viruses at an MOI of 0.5. The supernatant was collected at 24, 48, 72, and 96 h post-infection for TCID50 titration on Vero cells in triplicates. The graph was showed in mean log_10_TCID_50_/mL with a deviation and error bars represent the standard deviation of the mean (n = 3). rNDV; recombinant NDV.

S519G rNDV was produced and recovered to confirm the influence of the S519G mutant H domain on the growth kinetics of the rNDV replaced by the S519G mutant H domain. Based on the S519G mutant H domain, the serine sequence of the H domain was replaced with glycine via site-directed mutagenesis of the cDNA of the NDV genome (Fig. 4A). The cDNA genome in contact with the S519G mutant H domain was transfected with the modified Vaccinia virus as a helper virus and helper plasmids (pNP, pP, and pL) into HEp2 cells. The supernatant was then reinoculated into embryonated eggs. To remove the Vaccinia virus, more than three successive cultures of the recovered S519G mutant rNDV at the embryonated egg stage were used. The entirely removed Vaccinia virus was identified from the rNDV culture supernatant using PCR (Supplementary Data 3). Full genome sequence alignment of rNDV VG/GA and S519G mutant rNDV was performed (Supplementary data 4).

To assess the viral growth curve, Vero cells were infected with both rNDV and S519G mutant rNDV viruses, and the cell culture medium was collected at 24, 48, 72, and 96 h post-infection (h.p.i.). The viral titer in Vero cells was determined using a TCID_50_ assay. As shown in Fig. 4B, the rNDV titer gradually increased in a time-dependent manner. In Vero cells infected with rNDV, the viral titer peaked at 72 h.p.i. to a value of 10^7.33^ TCID_50_/mL, while in Vero cells infected with S519G mutant rNDV, the viral titer peaked at 48 h.p.i to 10^5.75^ TCID_50_/mL. These results suggest that mutations in the HN gene may affect the rate of virus growth or peak titer in Vero cells.

### Pathogenicity test of the S519G mutant rNDV

To determine whether the S519G mutant H domain affected the pathogenicity of the S519G mutant rNDV, the mean death time (MDT) (Supplementary Data 5) and intracerebral pathogenicity index (ICPI) (Supplementary Data 6) were measured. The S519G mutant rNDV average MDT and ICPI were 144 h and 0.34, respectively. The MDT of the S519G mutant rNDV belonged to the weakly pathogenic virus (lentogenic stain) and had a similar result to the original NDV MDT 140 h (no mutation in the H domain), showing the same result as that of the VG/GA strain. The ICPI of the S519G mutant rNDV was 0.34, which is a weakly pathogenic strain of NDV, and a similar ICPI number of rNDV was 0.36. MDT and ICPI results of the S519G mutant rNDV showed that the mutation at the 519 amino acid sequence did not influence the pathogenicity of NDV; it only influenced the cancer cell-specific affinity of NDV.

### Cancer cell killing effect of the S519G mutant rNDV

**Fig. 5:**
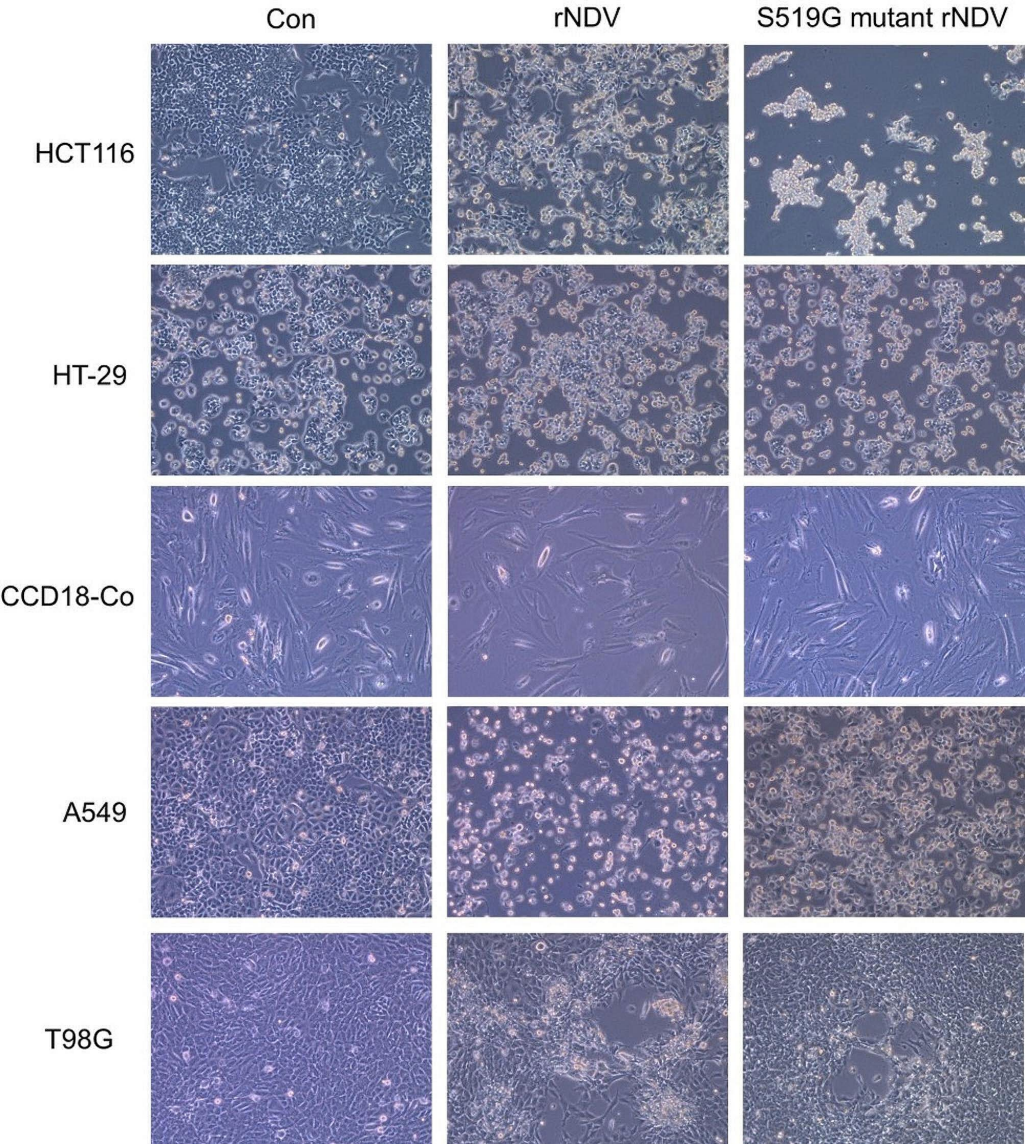
Cytopathic effect (CPE) in various cancer cells with S519G mutant rNDV virus. S519G mutant rNDV virus was infected in colorectal cancer cells (HCT 116 and HT-29), colorectal normal cells (CCD-18Co), and various cancer cells (T98G; glioblastoma, A549; lung cancer) at an MOI of 1. At 48 h post infection, there was no CPE in CCD-18Co, but CPE was observed in HCT 116 to form syncytia, HT-29 cells. In addition, the rNDV infection group was showed significant CPE in A519 cells but comparatively not in S519G mutant rNDV. rNDV; recombinant NDV

Various cancer cell-killing effects were investigated to demonstrate the effect of the rNDV substitution with the S519G H domain on oncolysis. The S519G rNDV mutant was inoculated into colorectal cancer cells (HCT 116, HT-29), normal colorectal cells (CCD-18Co), and other cancer cells (A547, lung cancer; T98G, glioblastoma). Cells were infected with the S519G mutant rNDV or the original rNDV as a positive control with a MOI of 1 at 48 h post-infection (Fig. 5). Cells infected with rNDV and the S519G mutant displayed a more irregular cellular morphology. Notably, HCT 116 cells infected with the S519G mutant rNDV showed the most pronounced irregular cellular morphology at 48 h.p.i. On the other hand, after observation for up to 96 h, weak cytopathic effects were observed on the CCD18-Co, A549, and T98G cells.

**Fig. 6:**
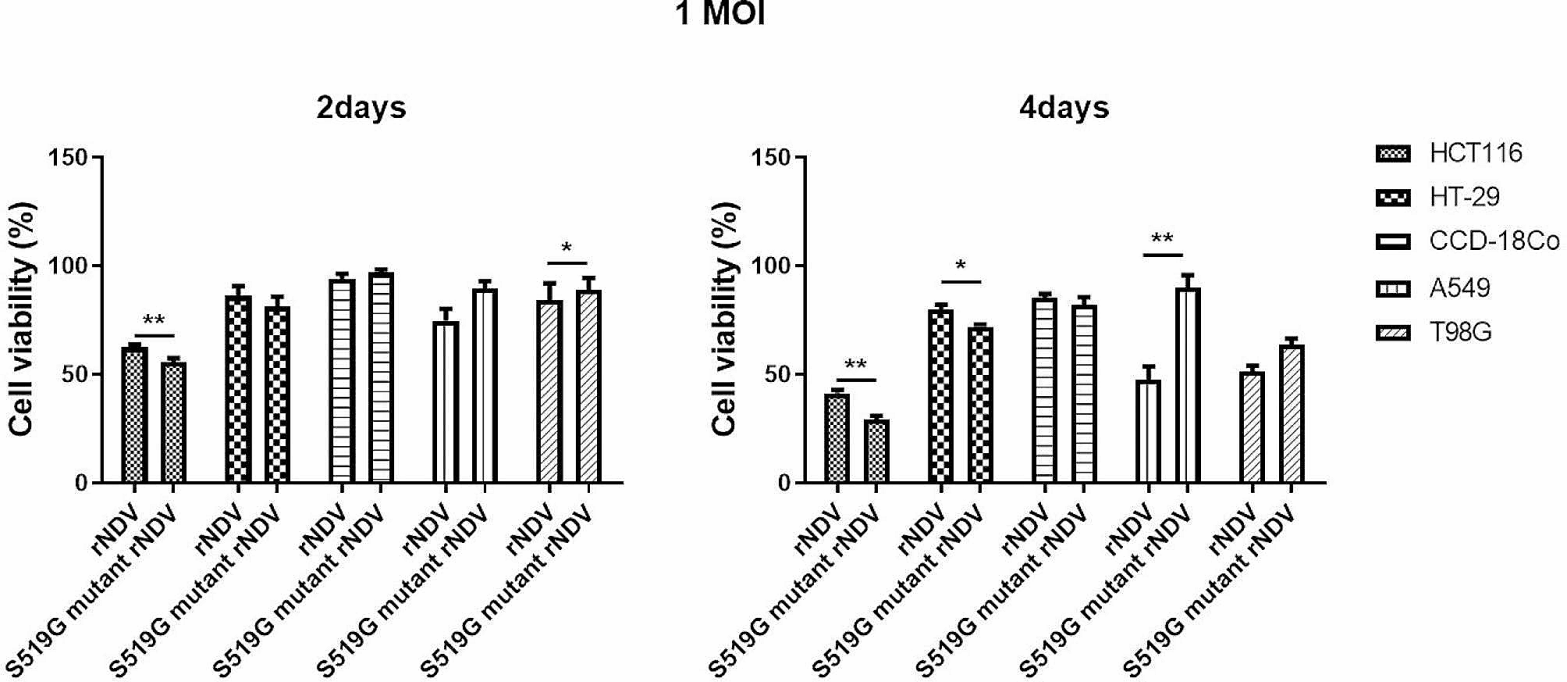
The susceptibility of colorectal cancer cells to rNDV or S519G mutant rNDV infections. MTT assays of rNDV and S519G mutant rNDV on colorectal cancer cells (HCT 116 and HT-29) colorectal normal cells (CCD-18Co), and various cancer cells (T98G; glioblastoma, A549; lung cancer). Before one day, the cells were cultured in 96-well tissue culture plates and infected with several titers (1 MOI) of rNDV or S519G mutant rNDV viruses. For negative control, the cells were grown in a culture medium. At 96 h post-infection, MTT assays were performed Cell Proliferation Assay kit. (n = 4, error bar: SEM, *p < 0.05, **p < 0.01, ***p < 0.001 determined by the Student’s t-test). MOI; multiplicity of infection, rNDV; recombinant NDV

To compare the oncolytic effects of rNDV and S519G mutant rNDV, an MTT assay was performed in the same cells as described above (Fig. 6). The results showed that the cell killing effect was directly proportional to the MOI of the virus (Supplementary Data 7). Nevertheless, the apoptotic effect of S519G mutant rNDV at an MOI of 1 was higher in HCT 116 and HT-29 cells than in A549 and T98G cells at 48 and 96 h.p.i. At 96 h.p.i., the percentages of apoptosis at 1 MOI for rNDV and S519G mutant rNDV were 41.287% and 29.13% (*p* < 0.01) in HCT 116 cells, 79.90% and 71.95% (*p* < 0.05) in HT-29 cells, 84.30% and 81.08% in CCD-18Co cells, 47.72% and 90.15% (*p* < 0.01) in A549 cells, and 41.46% and 51.27% (*p* < 0.05) in T98G cells, respectively. Interestingly, the oncolytic effect of the S519G mutant rNDV in A549 cells significantly decreased by 0.53-fold compared to that in rNDV infection and in HCT 116 cells significantly increased by 1.42-fold compared to that in rNDV infection. The S519G mutant rNDV virus’s binding affinity to HCT 116 colon cancer cells was improved. Interestingly, in other cancer cells (A549 and T98G), the oncolytic effect of normal rNDV was higher than that of the S519G mutant rNDV. These results revealed that the S519G mutant rNDV exhibited a significantly increased specific binding affinity and enhanced oncolytic effect against HCT 116 compared to other cancer cell lines.

### Tumor suppression effect of the S519G mutant rNDV in HCT 116, HT-29, and A549 xenograft mouse model

**Fig. 7:**
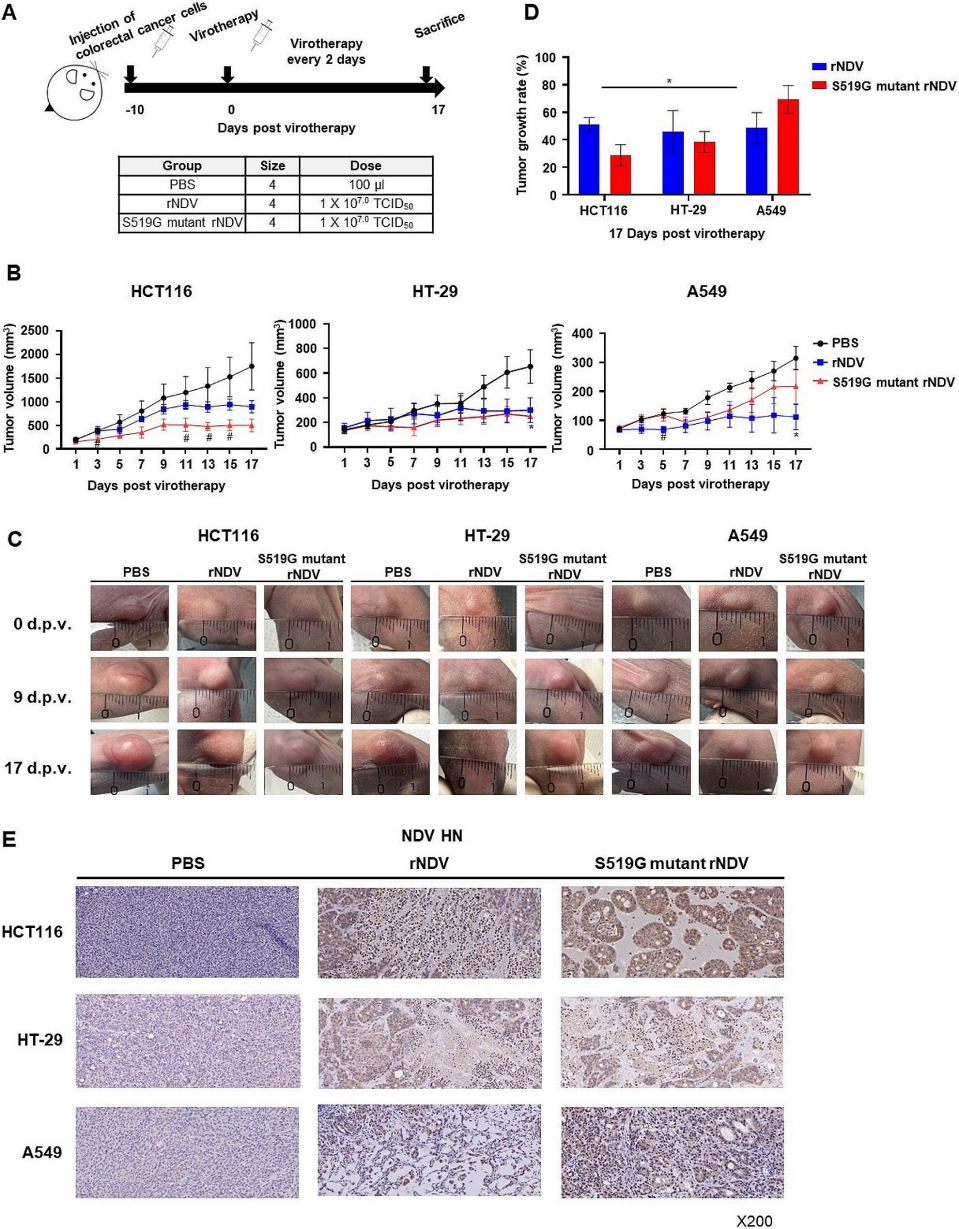
Tumor growth inhibition in a colorectal cancer and lung cancer xenograft mouse model. (**A**) A xenograft model was established to test the cancer tissue growth inhibitory effect of rNDV and S519G mutant rNDV viruses. 1 × 10^7^ cells/ml of HCT 116, HT-29, and A549 cells were inoculated into the left flank of the mouse. In each xenograft model, 12 SPF female BALB/c nude mice were randomly divided into groups with four mice per group (control, rNDV, and S519G mutant rNDV group). 10^7.0^ TCID_50_/dose of rNDV and S519G mutant rNDV viruses and PBS were treated by intravenous injection. Virus inoculation was repeated three times at 2-day intervals. (**B**) The tumor volume was observed at 1–17 days-post virotherapy in HCT 116, HT-29, and A549 xenograft mouse models (n = 4, error bar: SEM, *p < 0.05, **p < 0.01, ***p < 0.001 compared with control group, ^#^*p* < 0.05, ^##^*p* < 0.01, ^###^*p* < 0.001 compared with rNDV and S519G mutant rNDV group, and determined by the Student’s t-test). (**C**) Tumor size changes in mice were photographed after 0, 9, and 17 days-post virotherapy. (**D**) The tumor size of mice showed change (%) on the 17th days-post virotherapy based on the 1 day-post virotheray (n = 4, error bar: SEM, determined by the Student’s t-test). (**E**) Histology and immunohistochemistry of HCT116, HT-29, and A549 xenograft tumors. The representative images of immunohistochemistry for NDV HN are shown in brown. The rNDV; recombinant NDV

In the MTT assay, the S519G mutant of rNDV showed a more enhanced oncolytic effect than rNDV in HCT 116. To investigate the differences in the tumor suppression effect between rNDV and the S519G mutant rNDV, we constructed a xenograft model of mice transplanted with HCT 116 or HT-29 cells. In addition, an A549 xenograft mouse model was constructed and tested because, in A549 cells, a significantly enhanced cancer cell-killing effect was observed when they were infected with rNDV compared to S519G mutant rNDV. To compare the tumor-suppressive effect of S519G rNDV and rNDV, virotherapy was performed by intravenously injecting S519G rNDV or rNDV into BALB/c nu-/nu- mouse cancer cells implanted into the left flank (Fig. 7A). When the tumor volume reached 100–200 mm^3^ in HCT 116 and HT-29 cells and 50–100 mm^3^ in A549 cells, the mice were randomly divided into three groups of four mice and intravenously injected with 1 × 10^7.0^ TCID_50_/dose of either rNDV or S519G mutant rNDV every 2 d for 17 d. PBS was injected into the negative control group. Figure 7B and C show the tumor volumes of the mice. At 17 dpv, the tumor sizes in the PBS-injected group differed for all three cell types. HCT 116 (1747.5 mm^3^), followed by HT-29 (653.5 mm^3^) and A549 (314.2 mm^3^) cells. The tumor volume was observed in and compared between the S519G mutant rNDV group (496.2 mm^3^) and rNDV (891.5 mm^3^) or PBS (1747.5 mm^3^) groups in HCT 116 xenograft mice; the S519G mutant rNDV group (250.8 mm^3^) and rNDV (357.7 mm^3^) or PBS (653.5 mm^3^) groups in HT-29 xenograft mice; and the S519G mutant rNDV group (217.9 mm^3^) and rNDV (152.4 mm^3^) or PBS (314.2 mm^3^) groups in A549 xenograft mice at 17 d post-injection. The tumor suppression effect of the S519G rNDV- or rNDV inoculated group compared with the PBS inoculation group was 49.0% for the rNDV and 71.6% for the S519G mutant rNDV in HCT 116 cells, 54.0% for the rNDV and 61.6% for the S519G mutant rNDV in HT-29 cells, and 51.5% for the rNDV and 30.7% for the S519G mutant rNDV in A549 cells (Fig. 7D). S519G rNDV showed a significantly enhanced tumor-suppressive effect compared to rNDV in HCT 116 cell-transplanted mice; however, in the case of A549 cells, the tumor-suppressive effect of S519G rNDV was lower than that of rNDV. Tumors removed from mice inoculated with rNDV or S519G mutant rNDV investigated the expression levels of the HN protein of NDV using an IHC assay (Fig. 7E). Sections were prepared and stained with an anti-NDV HN antibody. In tumor sections intravenously inoculated with rNDV and S519G mutant rNDV, in all xenograft mouse models, rNDV HN was distributed throughout the tumor. In contrast, no virus was detected in the PBS group. More HN proteins were stained in the S519G mutant rNDV group than in the rNDV group in HCT116 cell-transplanted mice. Surprisingly, the number of stained HN proteins in HT-29 cell-transplanted animals was comparable across the rNDV and S519G mutant rNDV groups. Furthermore, the number of stained HN proteins was larger in the rNDV group than in the S519G mutant rNDV group in A549 cell-transfected mice. These findings demonstrated that S519G rNDV improved the tumor suppression effect specifically in HCT 116 cells and that this was due to the S519G H domain increasing the specific binding affinity to HCT 116 cell surface proteins.

## Discussion

Microbial infections, particularly viral infections, have been examined for their ability to inhibit tumor growth and regression. Viruses with therapeutic effects on tumors have been documented since the twentieth century and various viruses have been used for cancer treatment [6, 7]. Oncolytic viruses represent a new alternative approach to cancer therapy that involves the use of viruses to selectively target and destroy cancer cells while sparing healthy cells. The mechanism of action of oncolytic viruses involves a combination of direct virus-mediated destruction of cancer cells and activation of the immune system, resulting in a potent antitumor effect [48, 49]. Cancer cell-killing mechanisms mediated by viral infection and related genes or proteins have been discovered in more detail since the beginning of research on virus-based cancer treatment, and these discoveries provide the foundation for more efficient oncolytic virus development. Oncolytic viruses are being rapidly developed for cancer treatment. Oncolytic viruses are engineered or selected based on their ability to preferentially infect and replicate cancer cells. This selectivity is often achieved by modifying the virus to recognize specific molecular markers or receptors overexpressed on the surface of cancer cells. These modifications rendered the virus less likely to infect healthy cells. However, most oncolytic viruses are constructed with molecular-level modifications for cancer cells to selectively replicate but not for cancer cell-specific infections; they only block the propagation of normal cells. Therefore, only tumor-directed injectable oncolytic viruses have been commercialized for cancer treatment. Viral infection of the host cell initiates binding and interaction between a viral surface protein and a specific molecule on the cell surface. HER-2, CD155, CD40, and several other molecules are well-known proteins expressed on cancer cells and utilized as viral receptors for oncolytic virus binding and infection. However, molecular-level modifications of viral proteins that bind to cancer cell-specific surface proteins have not yet resulted in complete success [50]. NDV shows a wide range of tropism in mammalian cells [46, 51]. The HN protein-binding viral receptors in host cells are cell surface-anchoring glycoproteins. The glycoproteins on the cancer cell surface involved in NDV infection are unknown. Even if they originate from the same cancer type, the sialic acid pattern on the glycoproteins of cancer cells differs greatly depending on the cancer cell type and progression [52]. The HN protein has sialic acid-binding loops distributed throughout the hemagglutinin. Several studies have shown that specific amino acids play a role in sialic acid binding through non-covalent bonds [53]. Directed evolution has been used successfully for functional changes in proteins for specific purposes and is also a good tool for studying how amino acid sequence changes affect protein function and structure [54, 55]. Directed evolution can be used to alter the cancer cell specificity of oncolytic viruses. The HN protein of NDV is a good candidate receptor protein for directed evolution technology using the change in the specificity of NDV to cancer cells. The roles of HN proteins in the mechanism of NDV infection in host cells have been proposed in detail. HN is involved in receptor recognition, neuraminidase (NA) activity, and fusion. The HN protein recognizes sialic acid-conjugated protein receptors on cell surfaces. It promotes the fusion activity of the F protein, allowing viral and host cell membrane fusion, and NDV single-stranded RNA genomes to enter the cytoplasm of infected cells. HN is involved in sialic acid binding through specific amino acids positioned in the sialic acid-binding pocket of the globular head region in HN protein. Sialic acid binding converts the HN protein monomer into a dimer, resulting in the formation of new sialic acid-binding sites at the HN protein-dimer interface. Specific amino acid sequence loops are involved in the formation of additional sialic acid-binding sites at the HN dimeric interface. The S519G mutation occurs in the loop of two additional sialic acid-binding sites following HN dimer formation. Ser forms water-mediated hydrogen bonds with sialic acid [41]. Glycine is a simple amino acid that does not have a hydroxyl group as a side chain as serine does; however, it cannot be completely excluded that glycine cannot form a non-covalent bond with sialic acid. In addition to serine, several amino acids are involved in the hydrophobic pocket in the newly created sialic acid-binding site at the dimeric interface, and they also participate in non-covalent bonds with sialic acid. These amino acids were Gly169, Leu552, and Phe553 on one monomer and Phe156, Val517, and Leu561 on the other monomer. In binding the HN protein to sialic acid, creating a structural space, such as a hydrophobic pocket, is a very important prerequisite for forming an appropriate non-covalent bond with sialic acid. In particular, dimer and tetramer formation via sialic acid binding is essential for the multifunctionality of the HN protein. There are two possible changes in the binding of the HN protein to sialic acid due to glycine substitution for serine. Glycine increases the S519G mutant HN protein affinity for sialic acid of the same glycoprotein in HCT 116 cells. Another possible change is that the S519G mutant HN protein can bind to a new sialic acid of the glycoprotein of HCT 116 cells. Because the S519G mutant HN protein increased binding to HCT 116 cells but also decreased affinity to other cancer cells, it is not sufficient to explain the increase in specificity for HCT 116 cancer cells by increasing the affinity for the same sialic acid of the glycoprotein. Therefore, it can be postulated that the S519G mutant HN protein binds to a specific glycoprotein present only in HCT 116 colon cancer cells. More studies are needed to understand how glycine substitution for serine affects the S519G mutant HN protein and how glycine influences the dynamic structural changes and precise roles in binding to sialic acid. Glycine substitution with alanine in the amino acid sequence at position 319 improved the absorbance results of the ELISA by employing cell surface molecules from HCT 116 cells (data not shown). The 319 amino acid sequence is not found in the sialic acid-binding region, but it influences the binding affinity of the mutant H domain to glycoproteins of HCT 116 cancer cells. ELISA data from two separate amino acid substitutions revealed that amino acid sequence variations are either directly involved in the sialic acid-binding ability or are involved in protein structure modification. These findings indicate that it is still unclear which amino acid sequence changes may alter HN protein cancer cell selectivity.

In this study, the S519G mutant rNDV showed an increased cytotoxic effect on HCT 116 colorectal cancer cells (Fig. 6). Xenograft mouse model test results of S519G mutant rNDV showed better efficacy than rNDV in HCT 116 transplanted mice. But in the case of A549 lung cancer cells, xenograft model tests show that rNDV has better efficacy than S519G mutant rNDV. (Fig. 7). These results suggest that the enhanced tumor suppression effect of S519G mutant rNDV on HCT 116 cells is due to increased specificity for certain cancer cells rather than increased cytotoxicity. However, the glycoproteins on the HCT 116 cell surface that participate in binding with the S519G mutant HN protein of rNDV have not yet been discovered, and it is also unknown whether the S519G mutant HN protein is specific for the same glycoprotein or another glycoprotein on the surface of HCT 116 cells. The background to this elucidation is that the S519G mutant rNDV showed a significant decrease in virus proliferation in Vero cell culture, which is used for rNDV culture of host cells (Fig. 4B). However, the effect of the S519G mutant HN protein on the exact mechanism, such as the kinetics of the fusion process between the viral envelope and cell membrane or neuraminidase activity initiated by the S519G mutant HN protein-binding glycoprotein of the HCT 116 cell surface, remains unknown. All these will have to be revealed through further research, and we will experimentally determine which glycoprotein is involved in binding to the S519G mutant HN protein.

## Conclusions

This study provides insight into the discovery of a mutant recombinant virus with enhanced specificity for the surface proteins of cancer cells among the HN protein mutant library generated by the directed evolution technique, although further research is needed. This demonstrates the value of molecular modifications and screening methods for specific targeting of cancer cells.

### Electronic supplementary material

Below is the link to the electronic supplementary material.

Supplementary Material 1

Supplementary Material 2

Supplementary Material 3

Supplementary Material 4

Supplementary Material 5

Supplementary Material 6

Supplementary Material 7

Supplementary Material 8

## Data Availability

Not applicable.
