# Supplementary material for: The artificial amino acid change in the sialic acid-binding domain of the hemagglutinin neuraminidase of newcastle disease virus increases its specificity to HCT 116 colorectal cancer cells and tumor suppression effect
Source: Virol J. 2024 Jan 4;21:7. doi: 10.1186/s12985-023-02276-9 (PMC10768451; doi:10.1186/s12985-023-02276-9)
Supplement: Supplementary file 1 — Supplementary Material 1 [file 12985_2023_2276_MOESM1_ESM.pptx]

## Slide 1
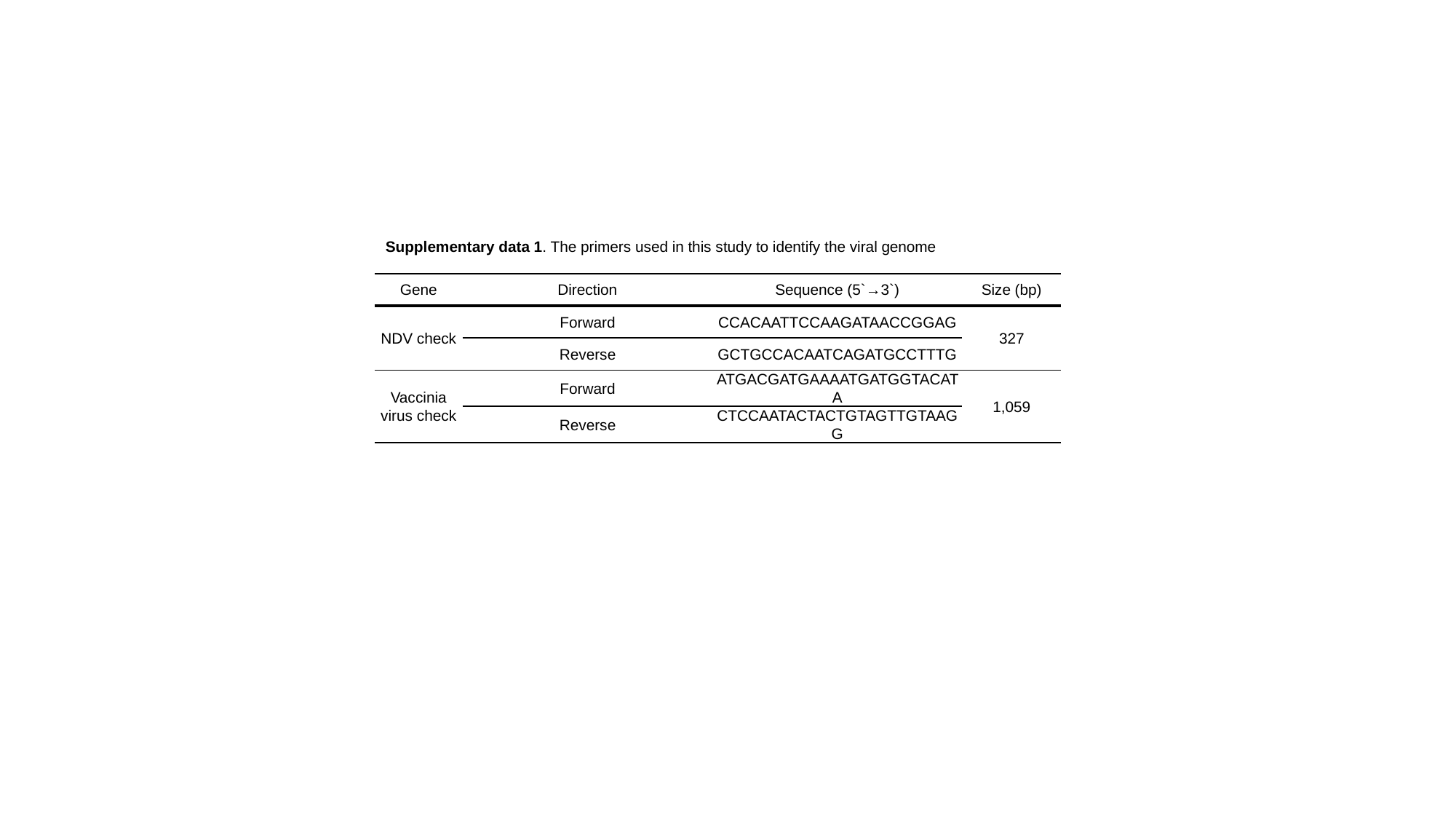

Supplementary data 1. The primers used in this study to identify the viral genome
| Gene | Direction | Sequence (5`→3`) | Size (bp) |
| --- | --- | --- | --- |
| NDV check | Forward | CCACAATTCCAAGATAACCGGAG | 327 |
| | Reverse | GCTGCCACAATCAGATGCCTTTG | |
| Vaccinia virus check | Forward | ATGACGATGAAAATGATGGTACATA | 1,059 |
| | Reverse | CTCCAATACTACTGTAGTTGTAAGG | |
