# Supplementary figures and images for: The artificial amino acid change in the sialic acid-binding domain of the hemagglutinin neuraminidase of newcastle disease virus increases its specificity to HCT 116 colorectal cancer cells and tumor suppression effect
Source: Virol J. 2024 Jan 4;21:7. doi: 10.1186/s12985-023-02276-9 (PMC10768451; doi:10.1186/s12985-023-02276-9)

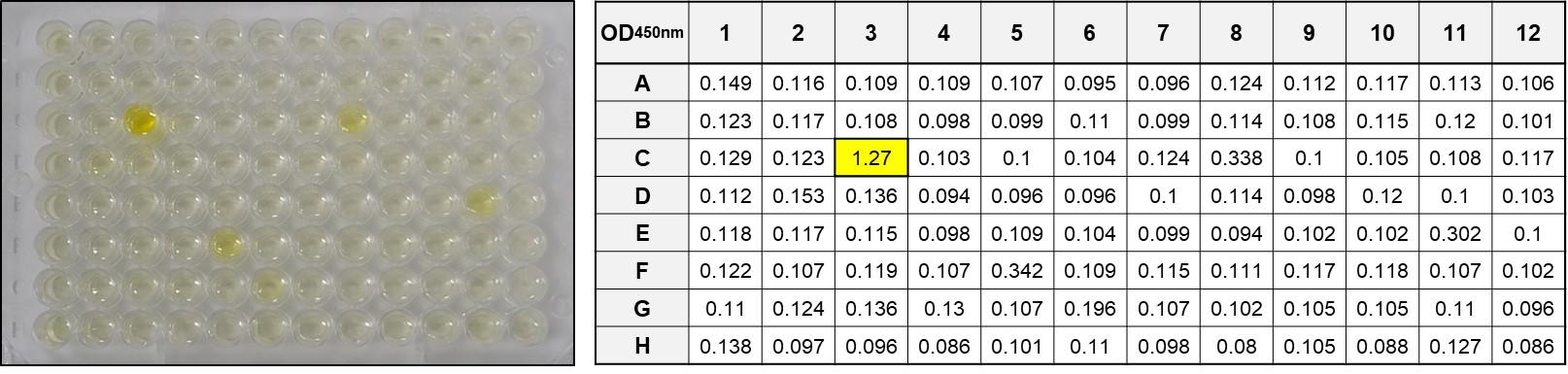

Supplement: Supplementary file 2 — Supplementary Material 2 [file 12985_2023_2276_MOESM2_ESM.jpg]

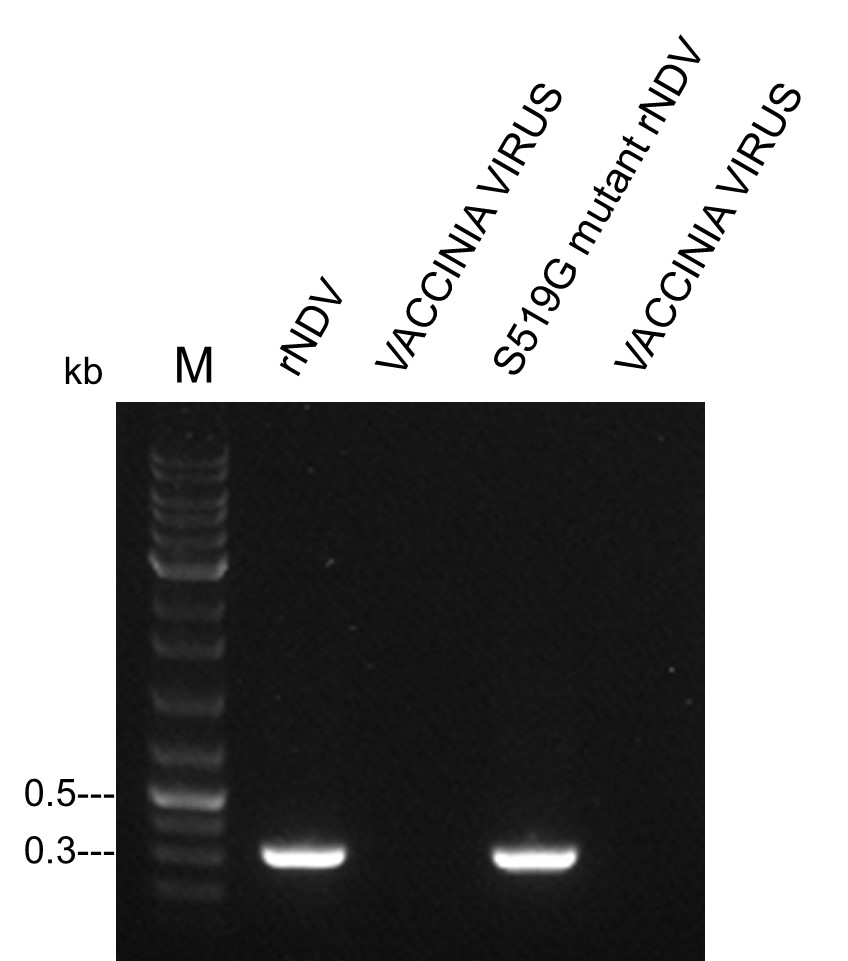

Supplement: Supplementary file 3 — Supplementary Material 3 [file 12985_2023_2276_MOESM3_ESM.jpg]

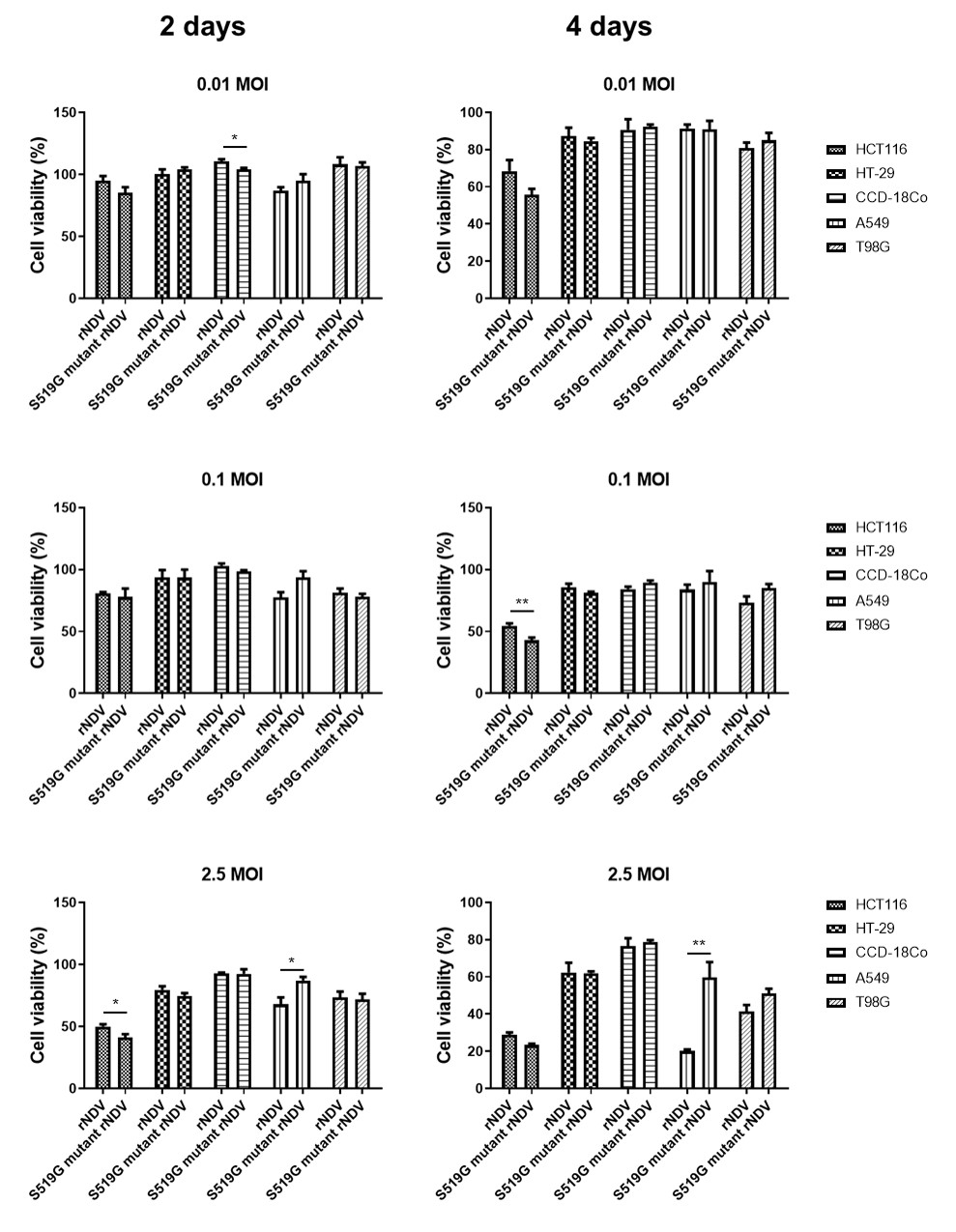

Supplement: Supplementary file 7 — Supplementary Material 7 [file 12985_2023_2276_MOESM7_ESM.jpg]

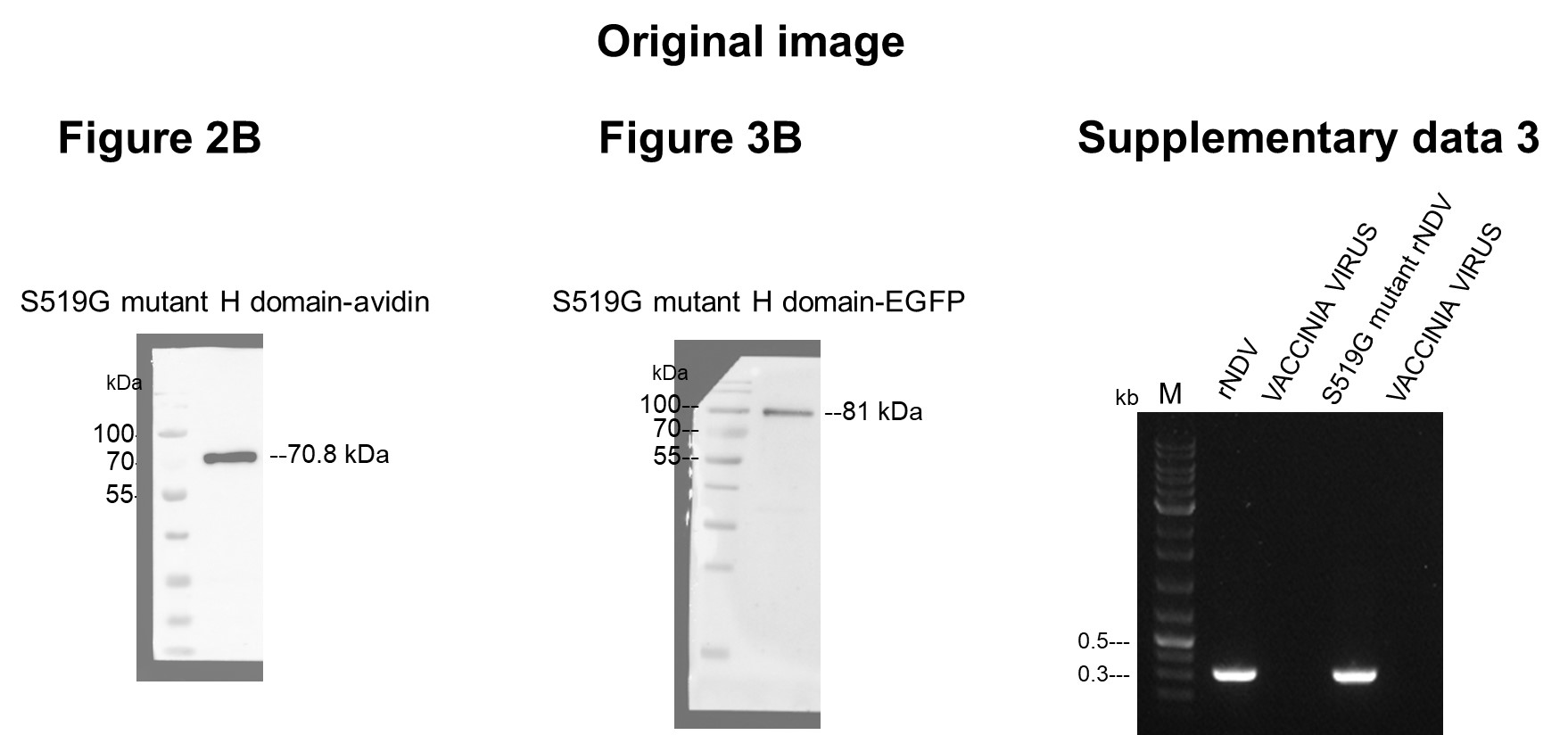

Supplement: Supplementary file 8 — Supplementary Material 8 [file 12985_2023_2276_MOESM8_ESM.jpg]
