# Supplementary material for: The artificial amino acid change in the sialic acid-binding domain of the hemagglutinin neuraminidase of newcastle disease virus increases its specificity to HCT 116 colorectal cancer cells and tumor suppression effect
Source: Virol J. 2024 Jan 4;21:7. doi: 10.1186/s12985-023-02276-9 (PMC10768451; doi:10.1186/s12985-023-02276-9)
Supplement: Supplementary file 4 — Supplementary Material 4 [file 12985_2023_2276_MOESM4_ESM.pptx]

## Slide 1
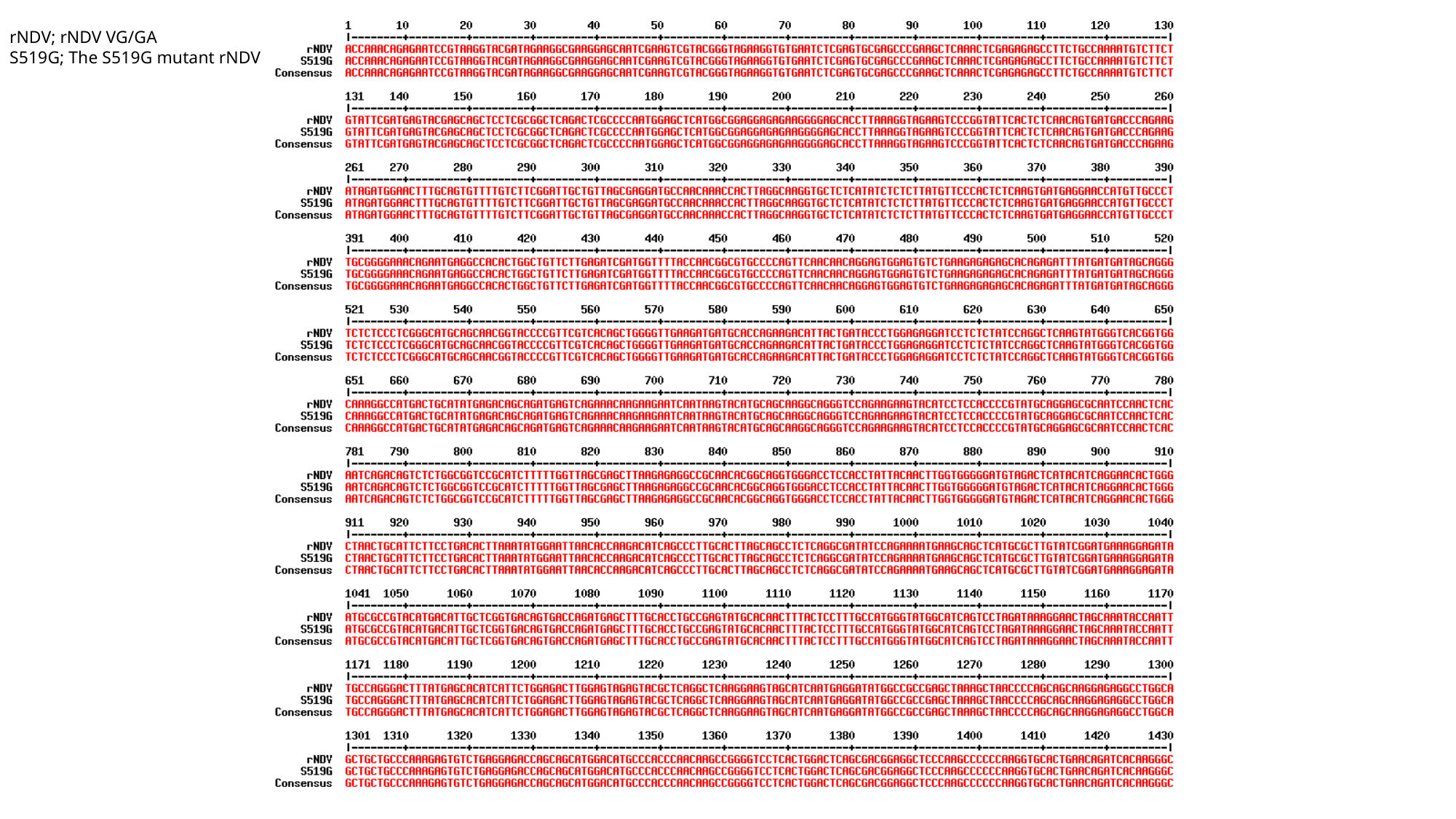

rNDV; rNDV VG/GA
S519G; The S519G mutant rNDV

## Slide 2
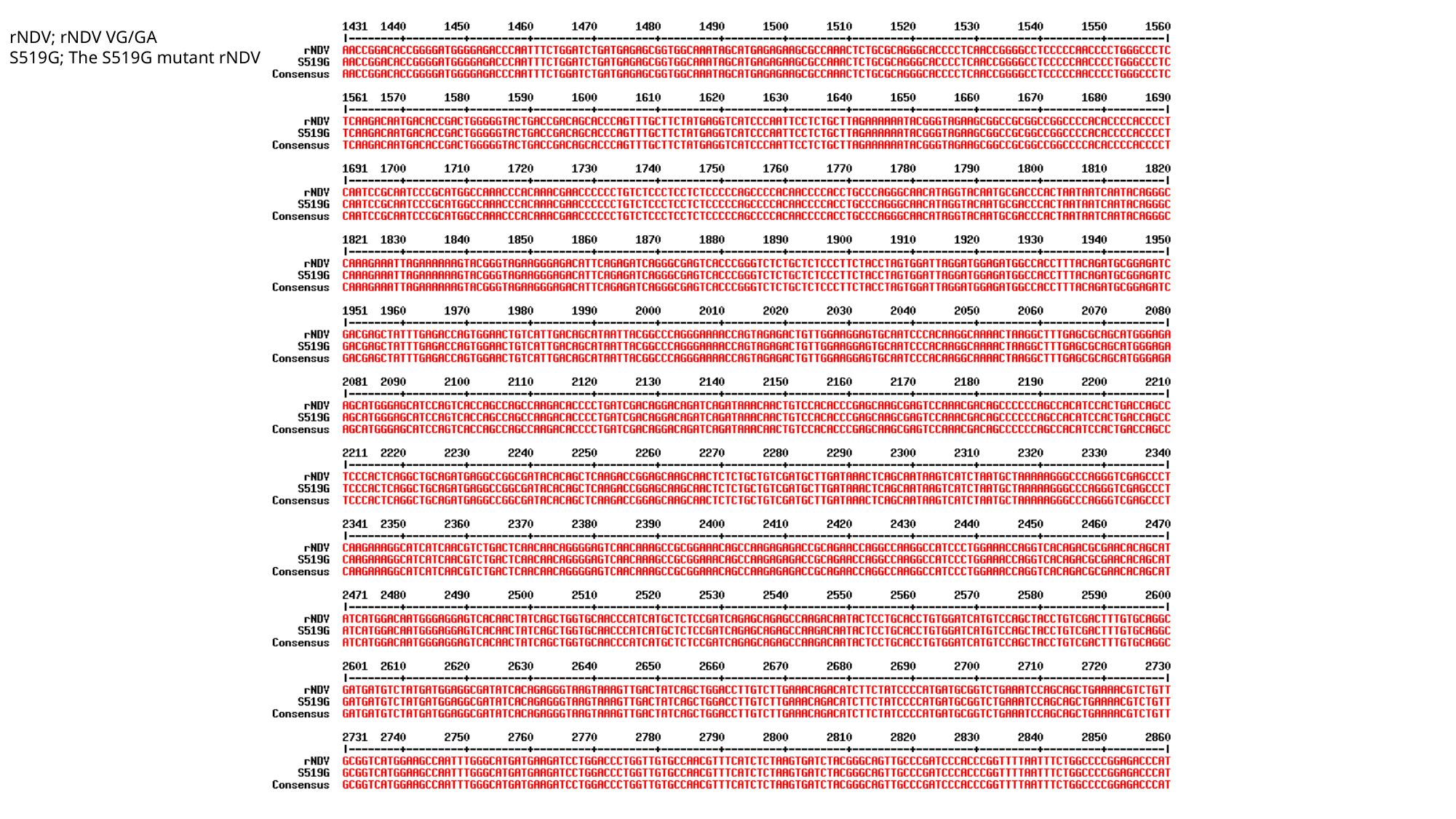

rNDV; rNDV VG/GA
S519G; The S519G mutant rNDV

## Slide 3
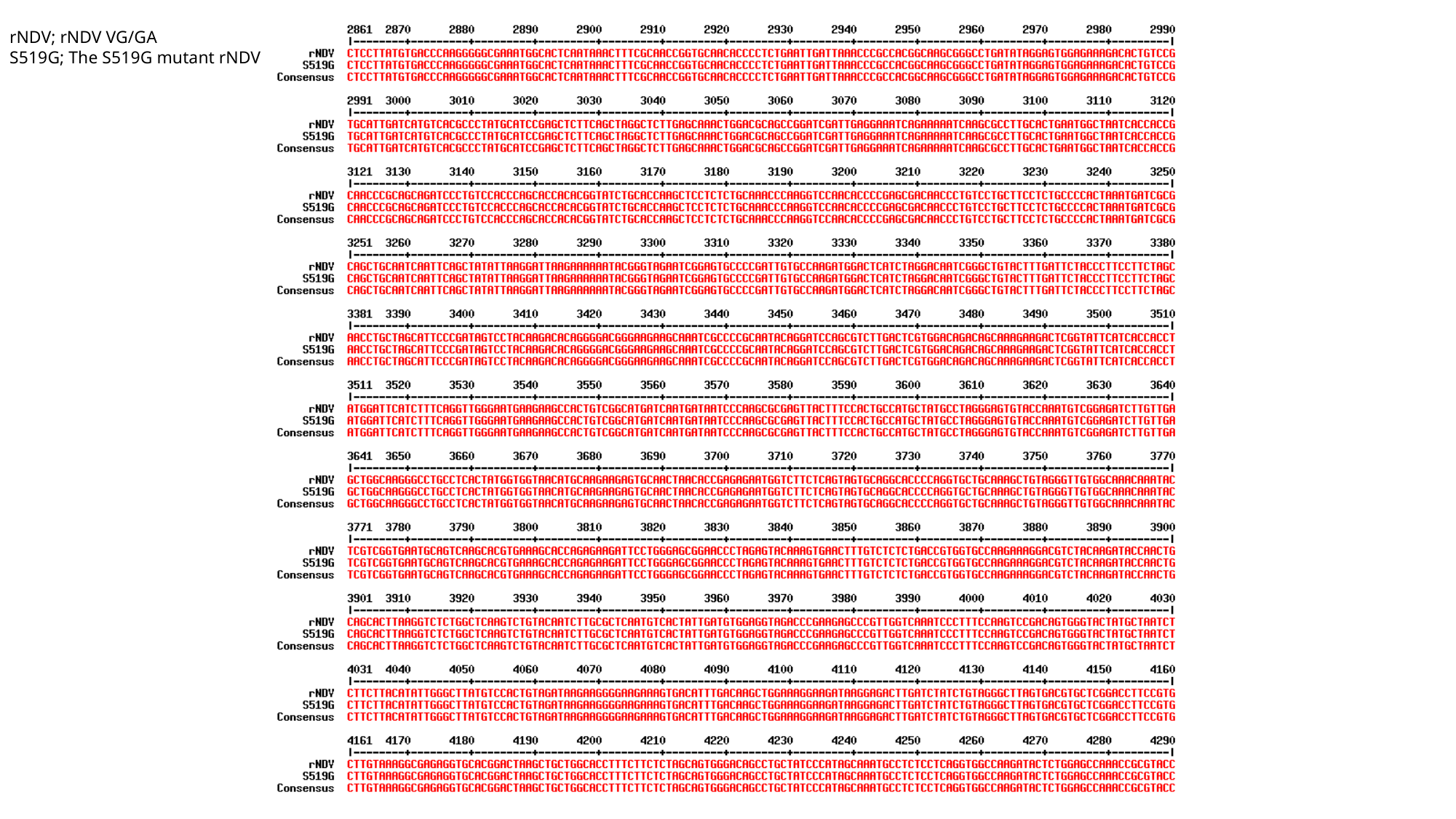

rNDV; rNDV VG/GA
S519G; The S519G mutant rNDV

## Slide 4
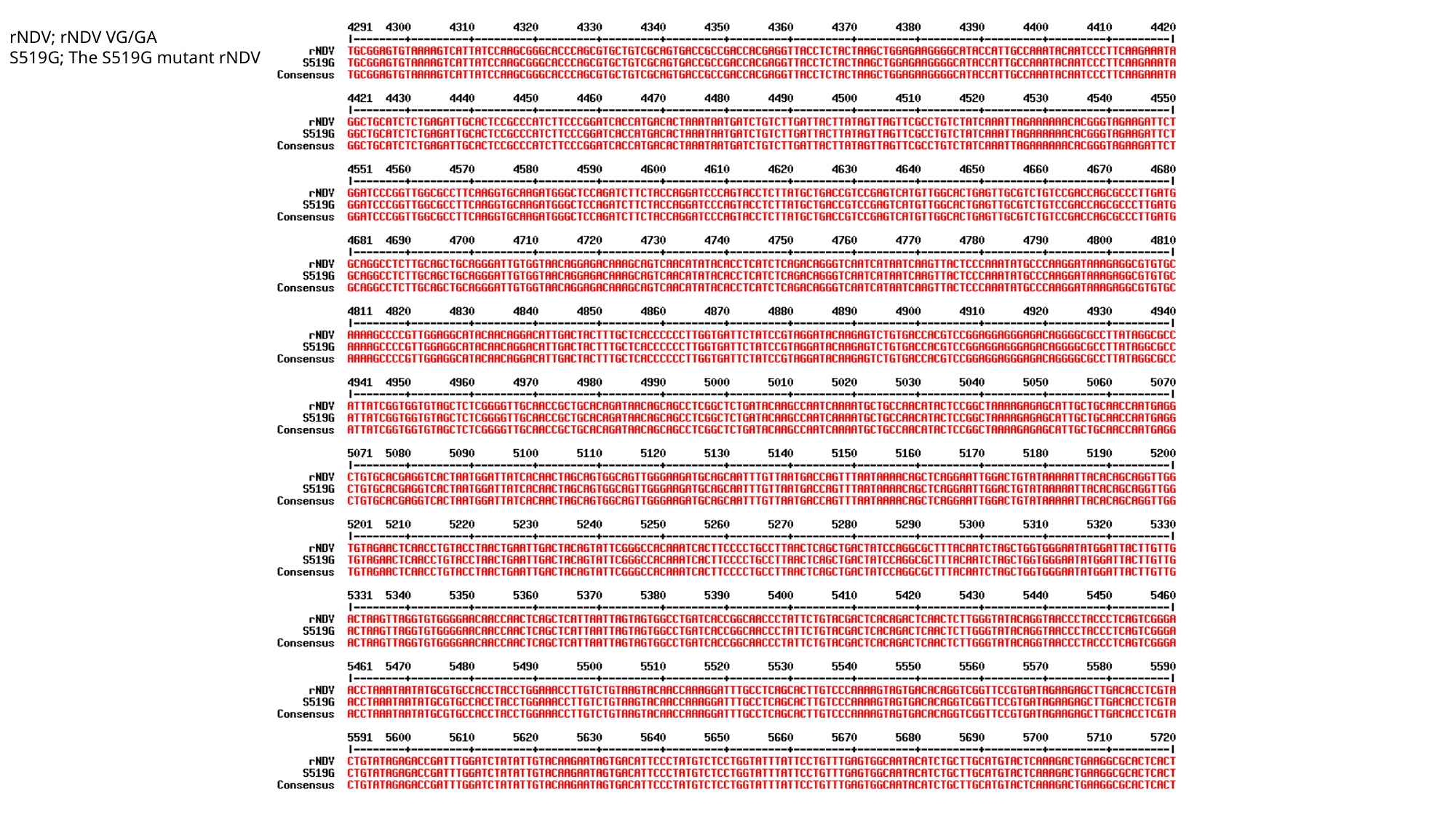

rNDV; rNDV VG/GA
S519G; The S519G mutant rNDV

## Slide 5
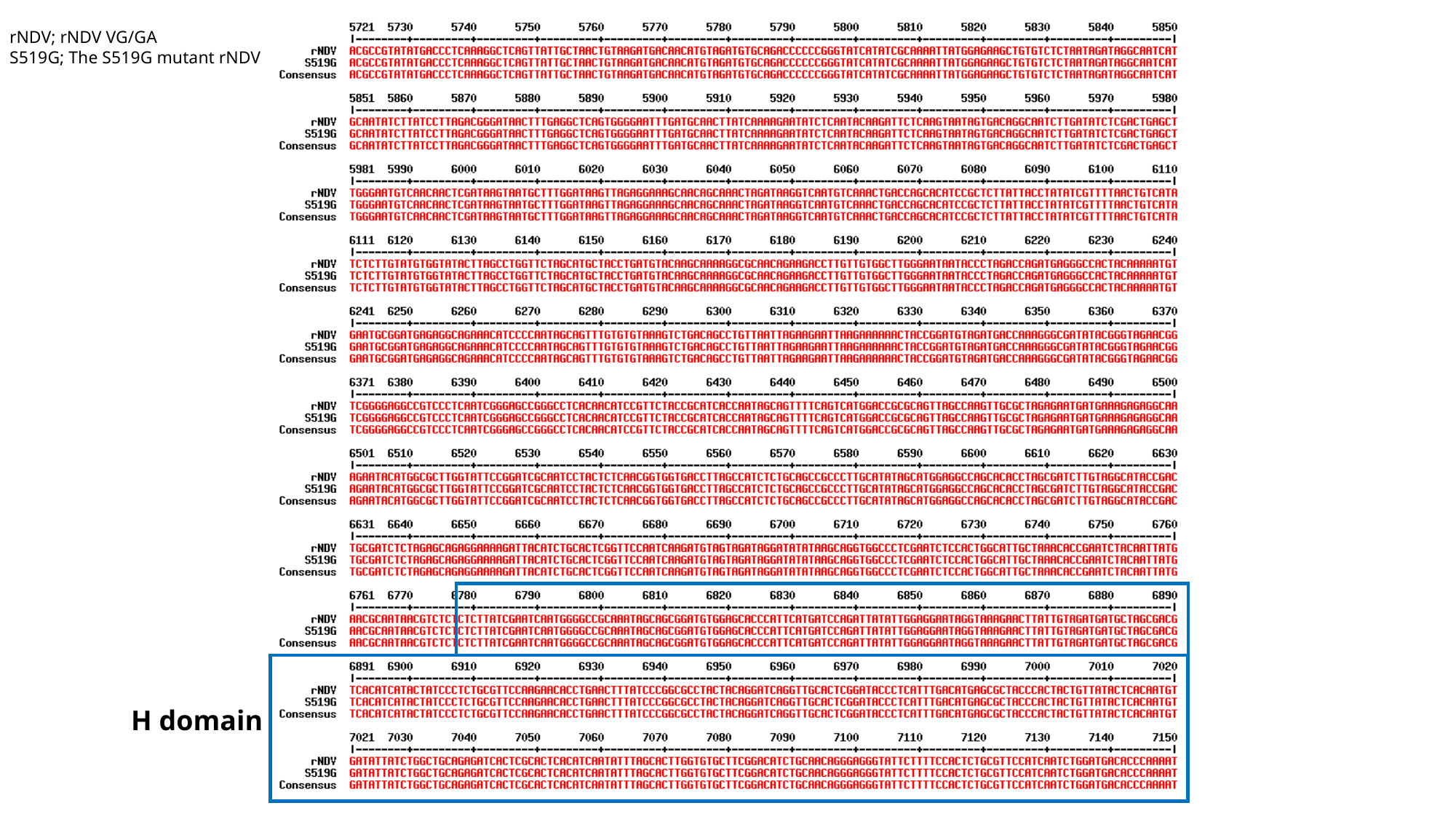

rNDV; rNDV VG/GA
S519G; The S519G mutant rNDV
H domain

## Slide 6
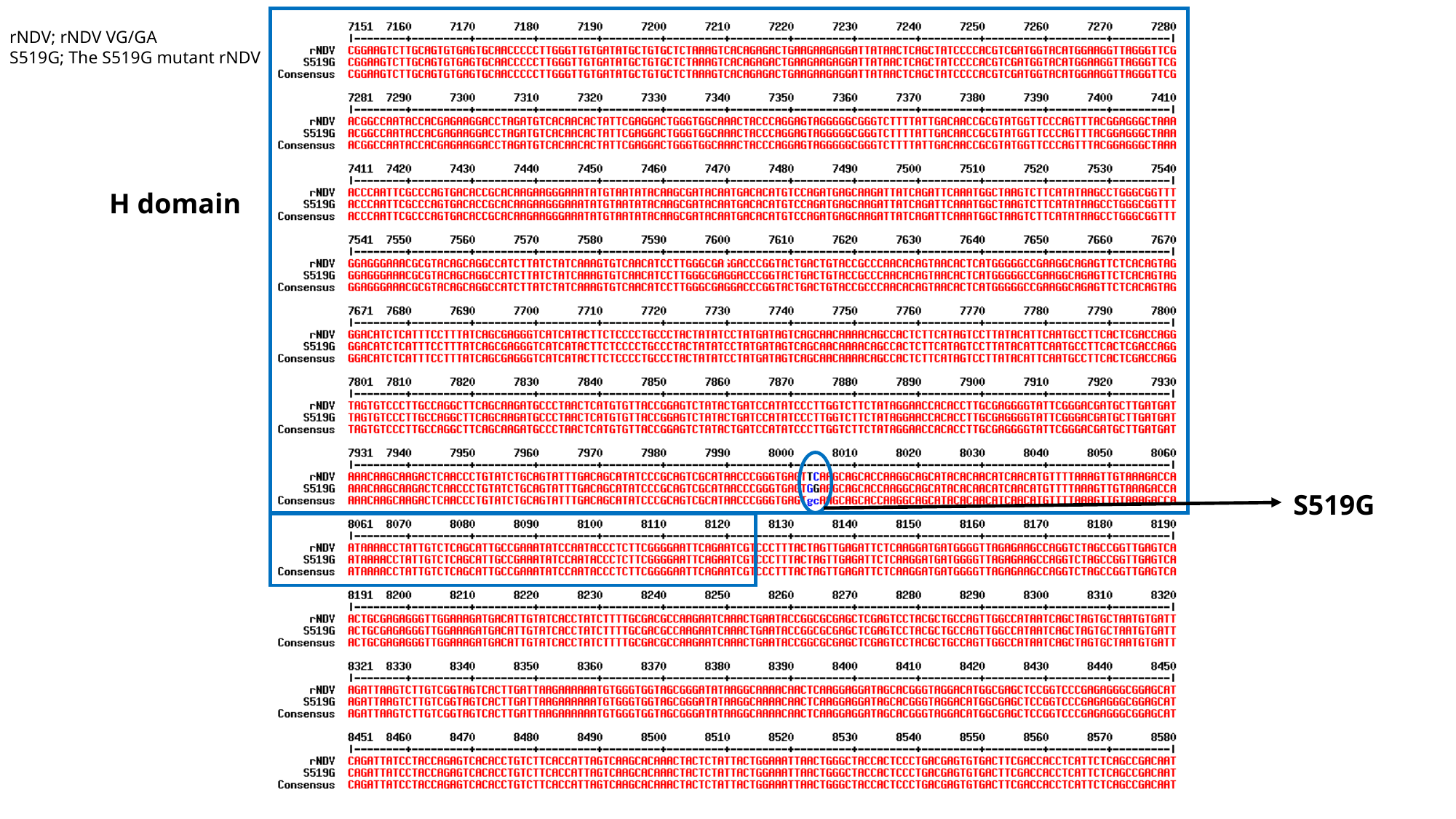

c
rNDV; rNDV VG/GA
S519G; The S519G mutant rNDV
H domain
S519G

## Slide 7
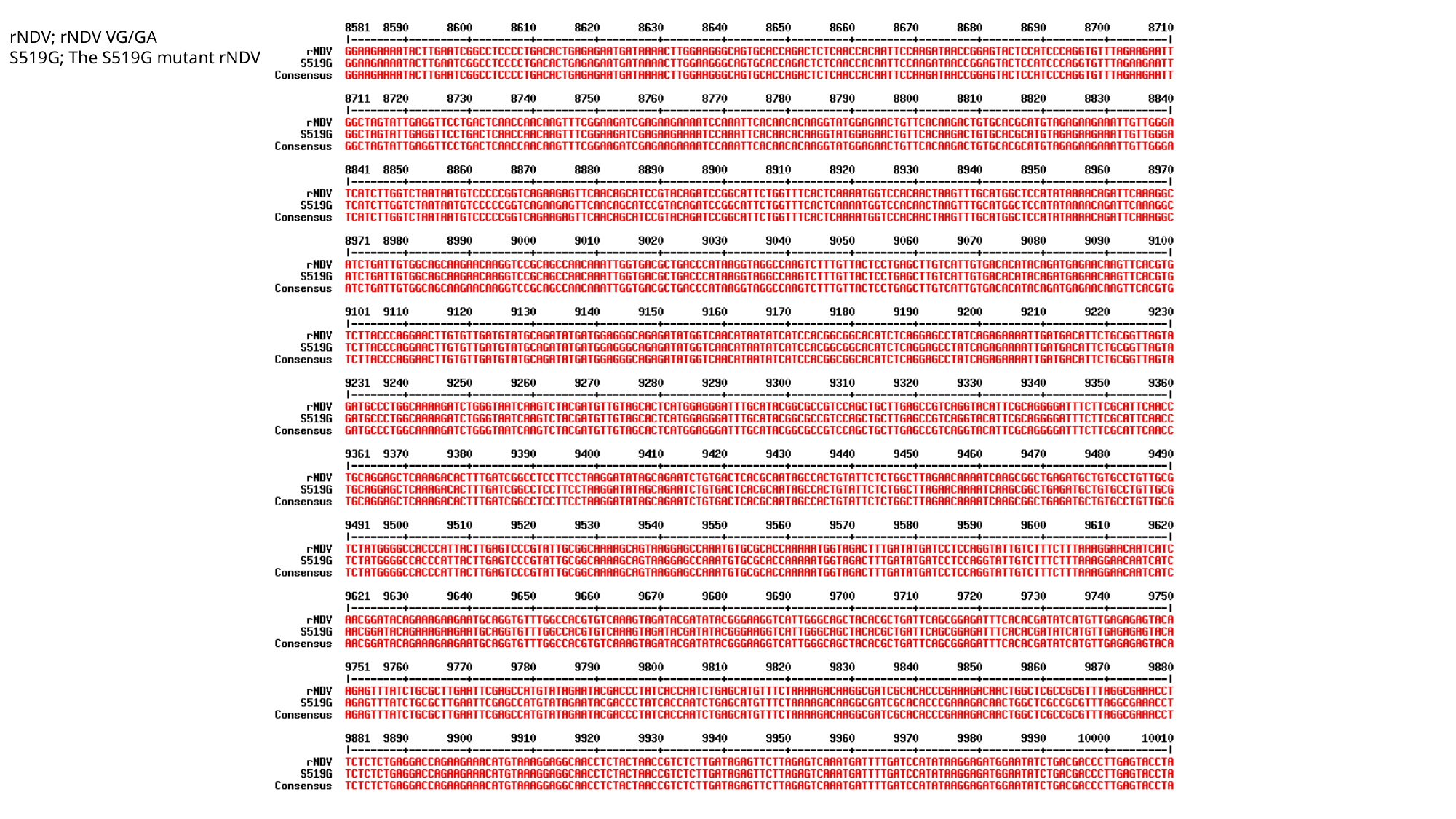

rNDV; rNDV VG/GA
S519G; The S519G mutant rNDV

## Slide 8
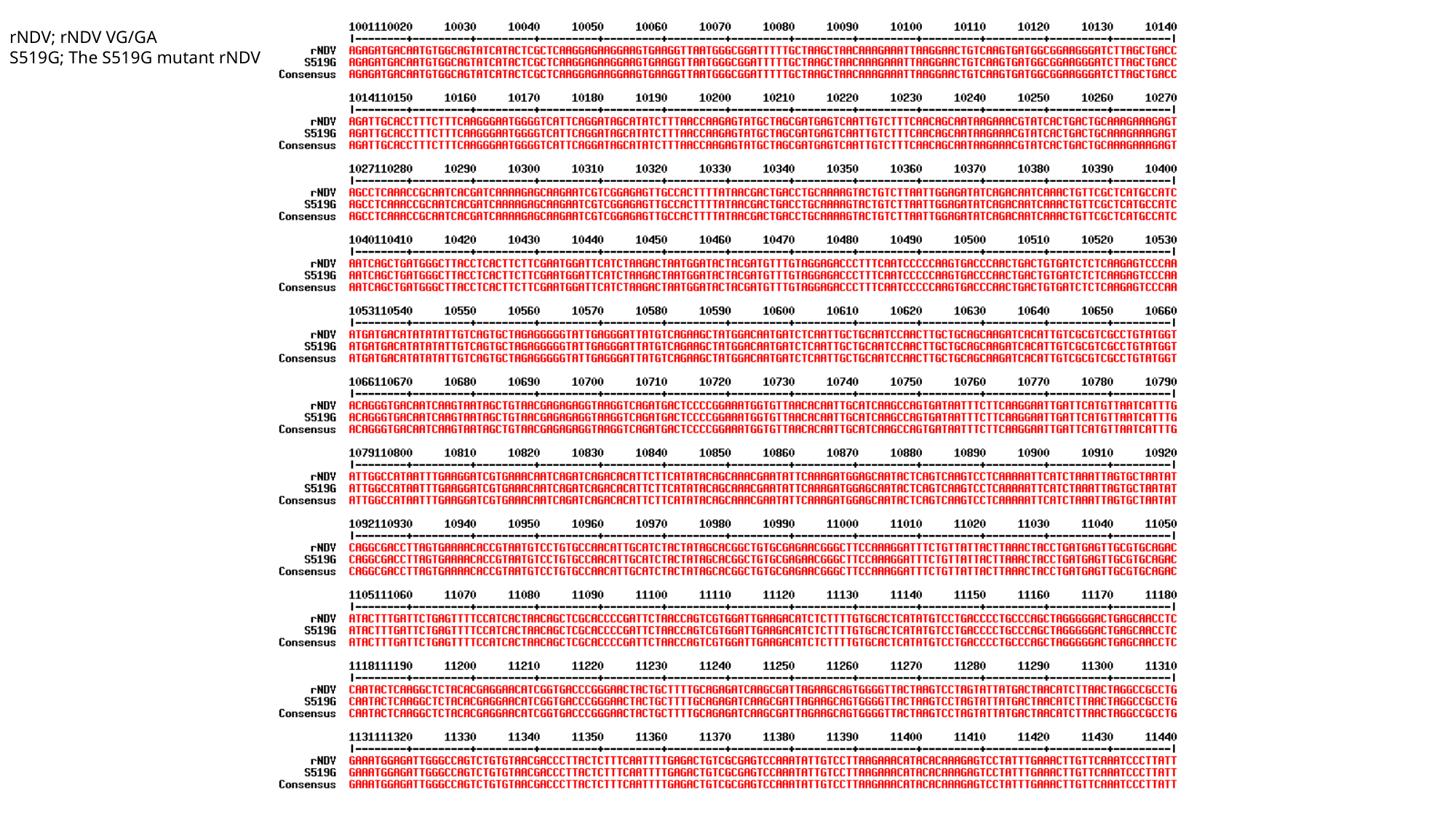

rNDV; rNDV VG/GA
S519G; The S519G mutant rNDV

## Slide 9
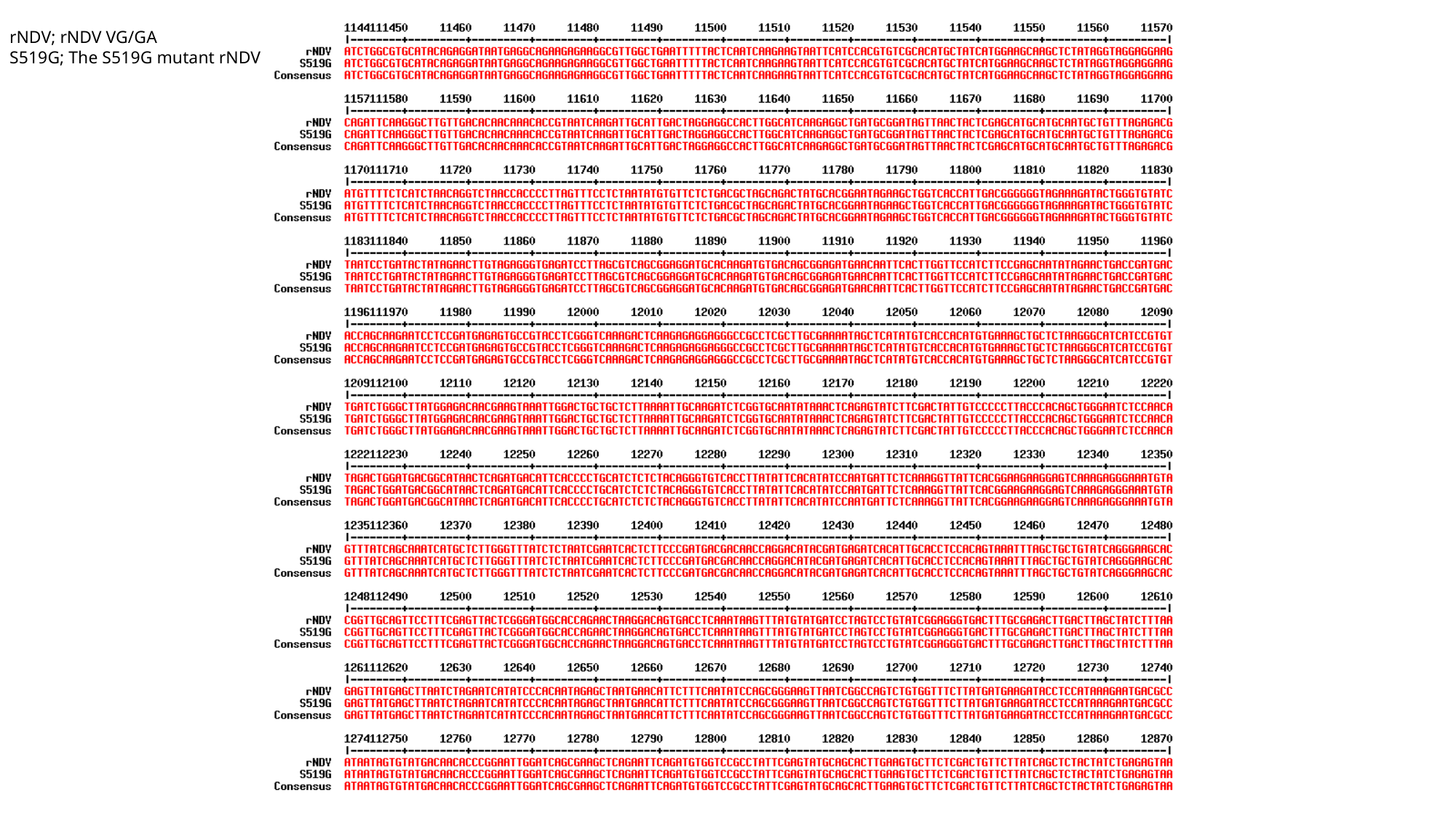

rNDV; rNDV VG/GA
S519G; The S519G mutant rNDV

## Slide 10
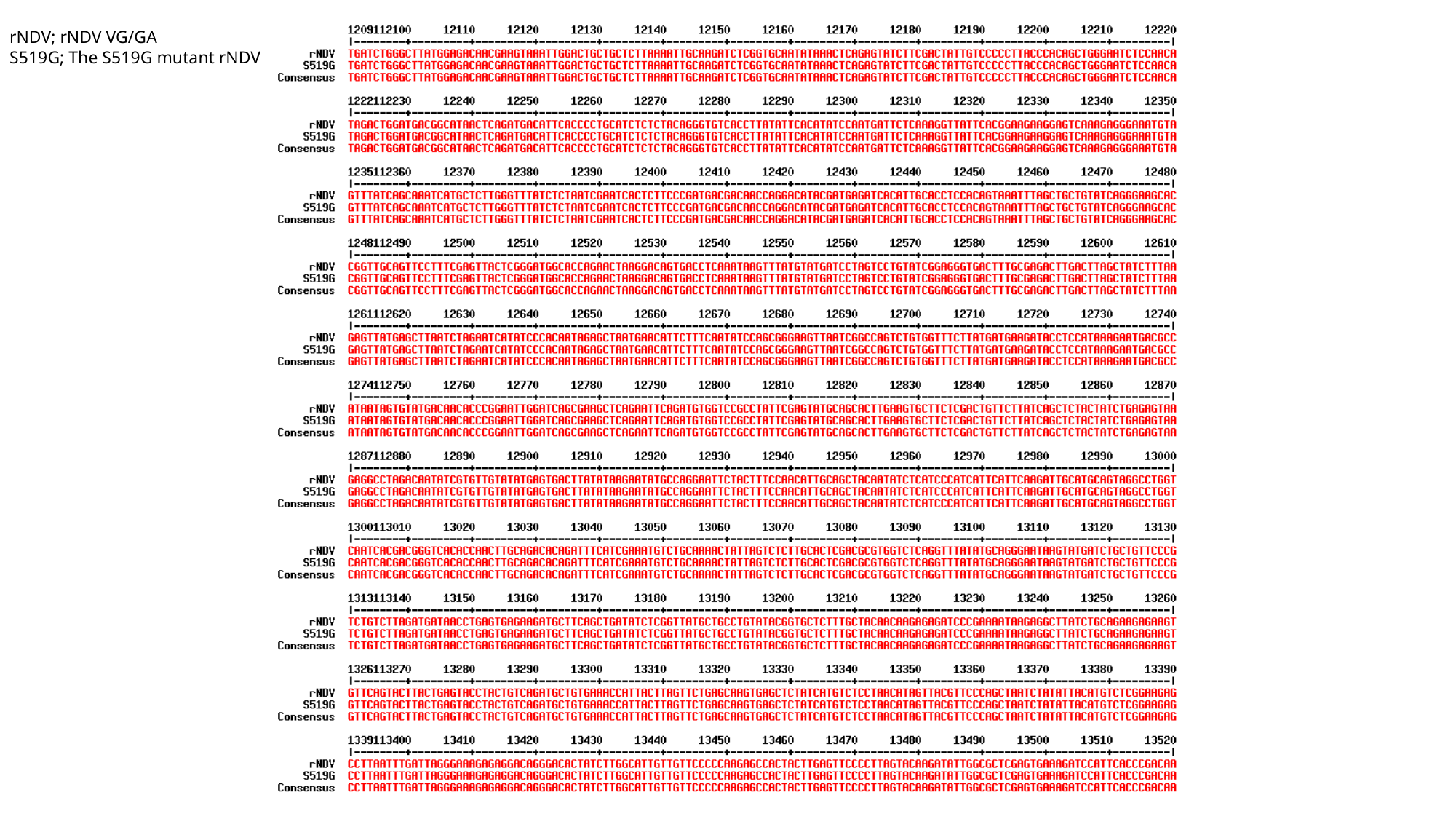

rNDV; rNDV VG/GA
S519G; The S519G mutant rNDV

## Slide 11
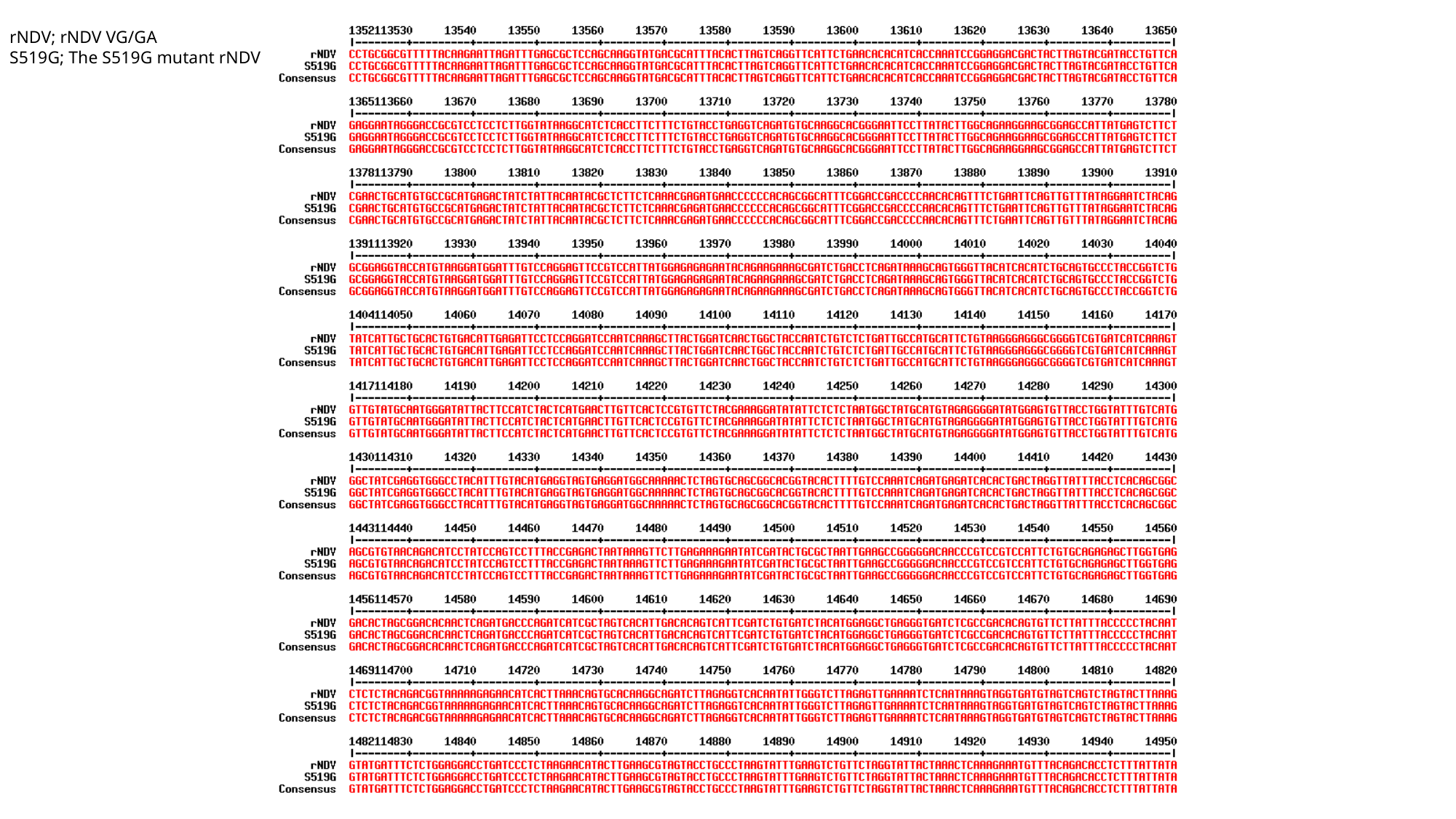

rNDV; rNDV VG/GA
S519G; The S519G mutant rNDV

## Slide 12
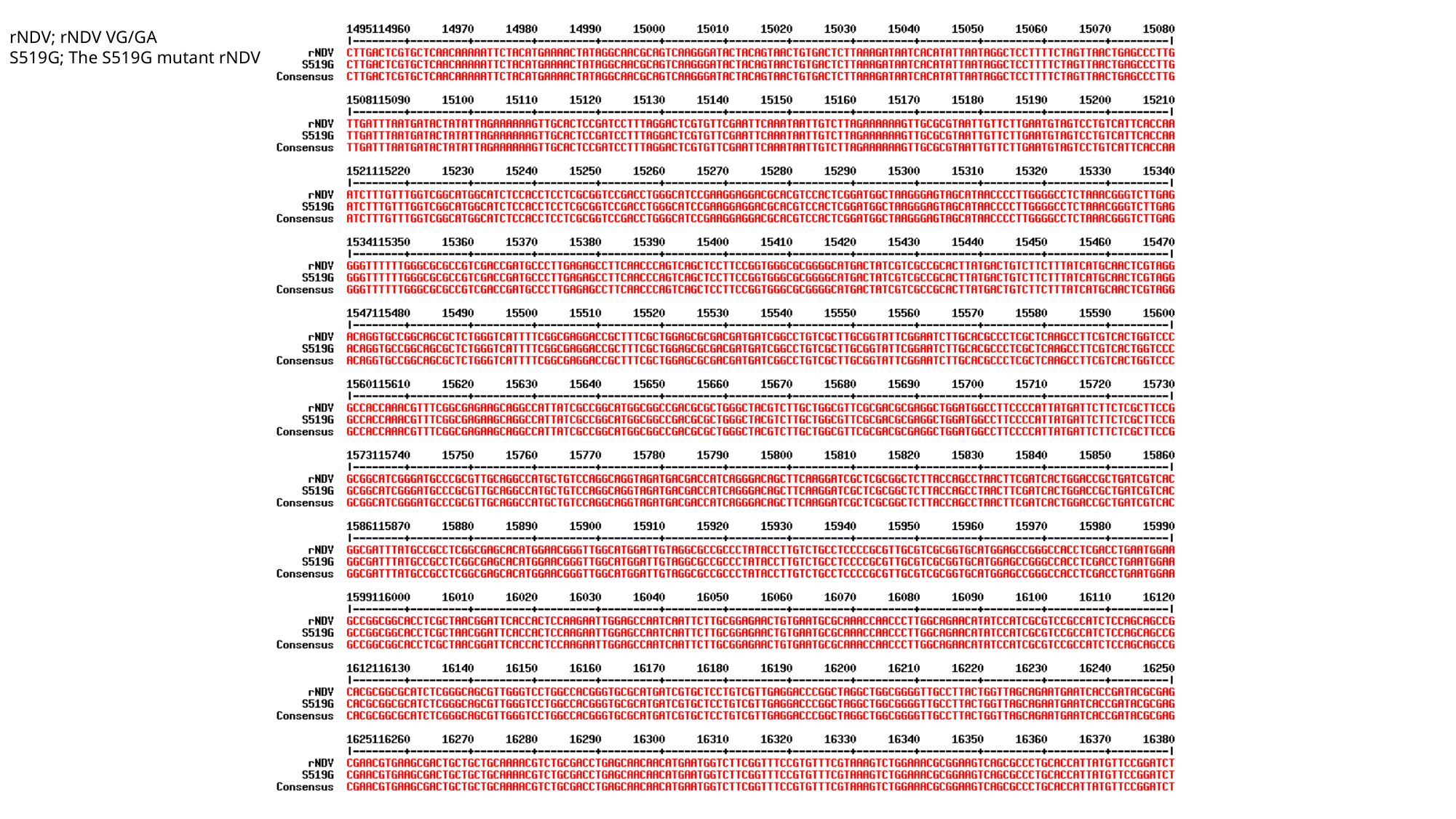

rNDV; rNDV VG/GA
S519G; The S519G mutant rNDV

## Slide 13
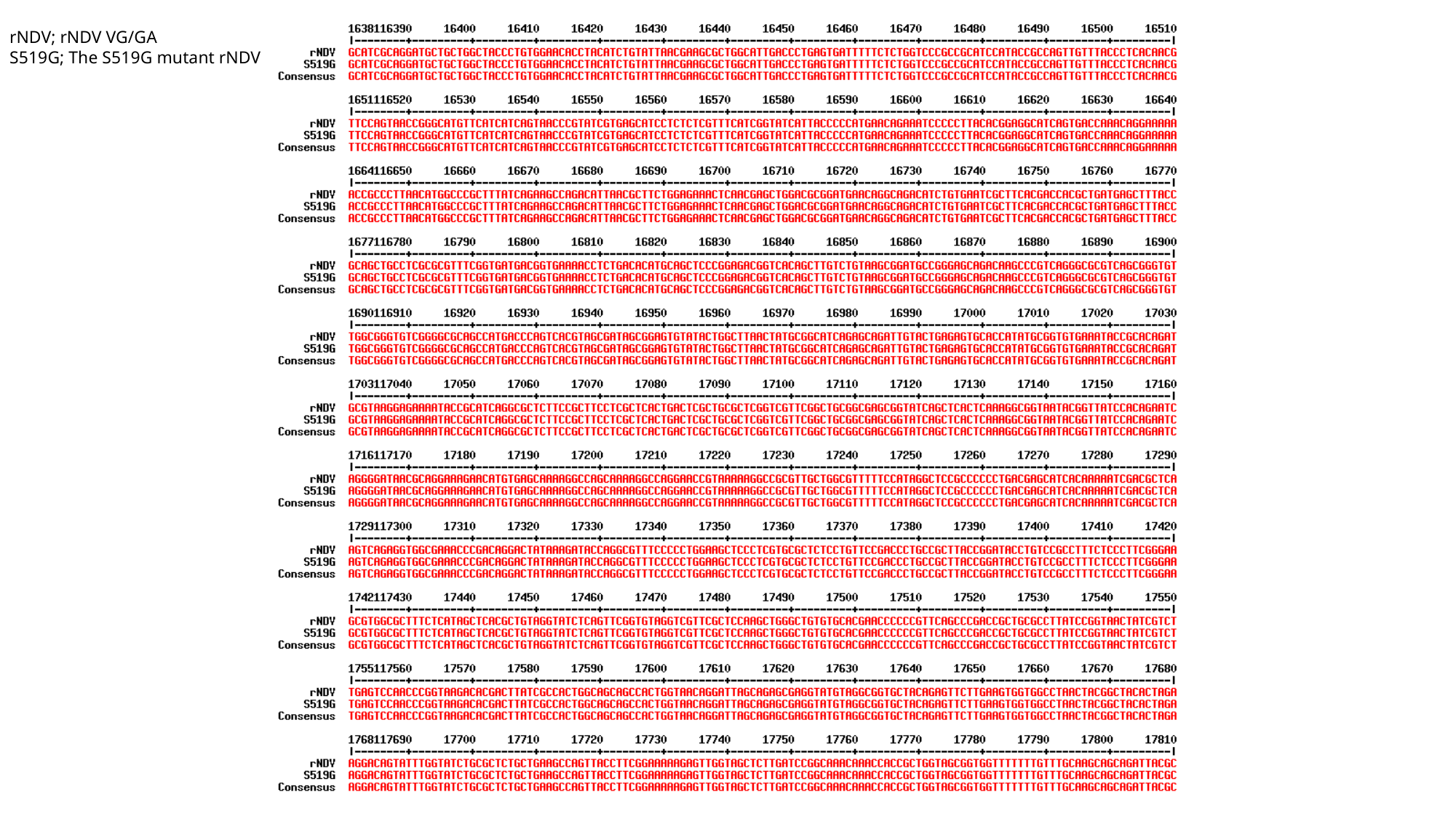

rNDV; rNDV VG/GA
S519G; The S519G mutant rNDV

## Slide 14
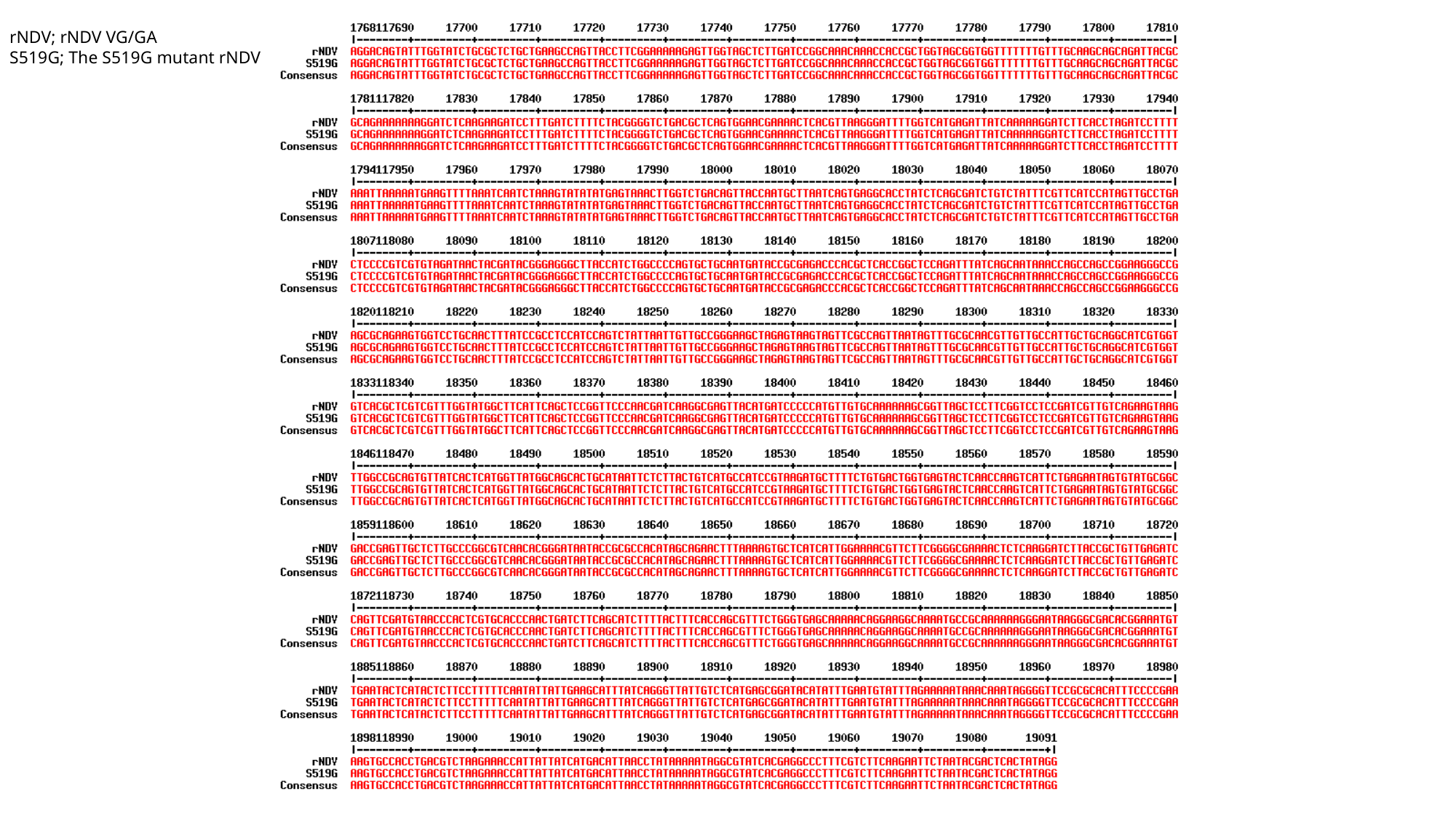

rNDV; rNDV VG/GA
S519G; The S519G mutant rNDV
