# Supplementary material for: The artificial amino acid change in the sialic acid-binding domain of the hemagglutinin neuraminidase of newcastle disease virus increases its specificity to HCT 116 colorectal cancer cells and tumor suppression effect
Source: Virol J. 2024 Jan 4;21:7. doi: 10.1186/s12985-023-02276-9 (PMC10768451; doi:10.1186/s12985-023-02276-9)
Supplement: Supplementary file 5 — Supplementary Material 5 [file 12985_2023_2276_MOESM5_ESM.pptx]

## Slide 1
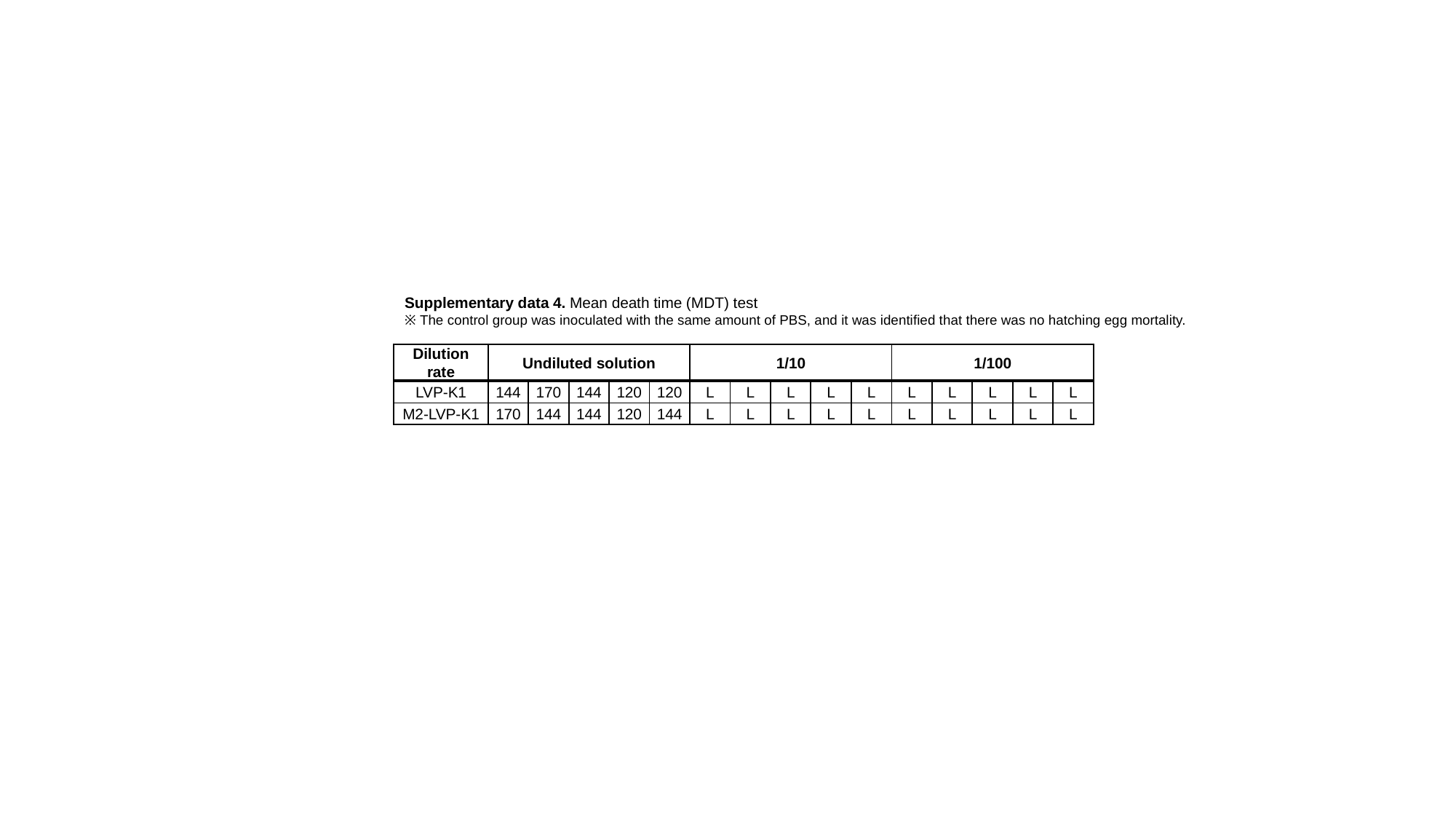

Supplementary data 4. Mean death time (MDT) test
※ The control group was inoculated with the same amount of PBS, and it was identified that there was no hatching egg mortality.
| Dilution rate | Undiluted solution | | | | | 1/10 | | | | | 1/100 | | | | |
| --- | --- | --- | --- | --- | --- | --- | --- | --- | --- | --- | --- | --- | --- | --- | --- |
| LVP-K1 | 144 | 170 | 144 | 120 | 120 | L | L | L | L | L | L | L | L | L | L |
| M2-LVP-K1 | 170 | 144 | 144 | 120 | 144 | L | L | L | L | L | L | L | L | L | L |
