# Supplementary material for: The artificial amino acid change in the sialic acid-binding domain of the hemagglutinin neuraminidase of newcastle disease virus increases its specificity to HCT 116 colorectal cancer cells and tumor suppression effect
Source: Virol J. 2024 Jan 4;21:7. doi: 10.1186/s12985-023-02276-9 (PMC10768451; doi:10.1186/s12985-023-02276-9)
Supplement: Supplementary file 6 — Supplementary Material 6 [file 12985_2023_2276_MOESM6_ESM.pptx]

## Slide 1
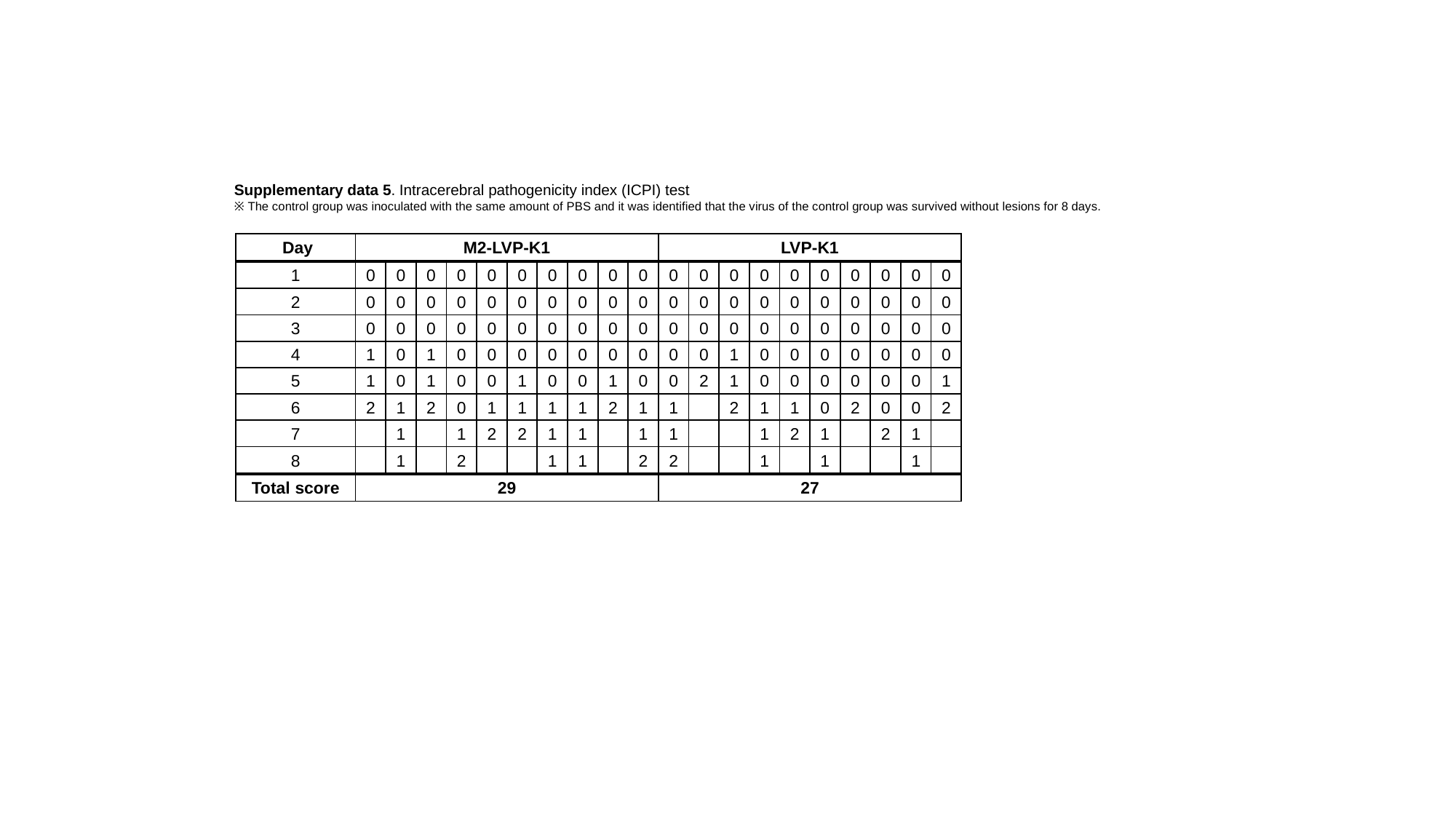

Supplementary data 5. Intracerebral pathogenicity index (ICPI) test
※ The control group was inoculated with the same amount of PBS and it was identified that the virus of the control group was survived without lesions for 8 days.
| Day | M2-LVP-K1 | | | | | | | | | | LVP-K1 | | | | | | | | | |
| --- | --- | --- | --- | --- | --- | --- | --- | --- | --- | --- | --- | --- | --- | --- | --- | --- | --- | --- | --- | --- |
| 1 | 0 | 0 | 0 | 0 | 0 | 0 | 0 | 0 | 0 | 0 | 0 | 0 | 0 | 0 | 0 | 0 | 0 | 0 | 0 | 0 |
| 2 | 0 | 0 | 0 | 0 | 0 | 0 | 0 | 0 | 0 | 0 | 0 | 0 | 0 | 0 | 0 | 0 | 0 | 0 | 0 | 0 |
| 3 | 0 | 0 | 0 | 0 | 0 | 0 | 0 | 0 | 0 | 0 | 0 | 0 | 0 | 0 | 0 | 0 | 0 | 0 | 0 | 0 |
| 4 | 1 | 0 | 1 | 0 | 0 | 0 | 0 | 0 | 0 | 0 | 0 | 0 | 1 | 0 | 0 | 0 | 0 | 0 | 0 | 0 |
| 5 | 1 | 0 | 1 | 0 | 0 | 1 | 0 | 0 | 1 | 0 | 0 | 2 | 1 | 0 | 0 | 0 | 0 | 0 | 0 | 1 |
| 6 | 2 | 1 | 2 | 0 | 1 | 1 | 1 | 1 | 2 | 1 | 1 | | 2 | 1 | 1 | 0 | 2 | 0 | 0 | 2 |
| 7 | | 1 | | 1 | 2 | 2 | 1 | 1 | | 1 | 1 | | | 1 | 2 | 1 | | 2 | 1 | |
| 8 | | 1 | | 2 | | | 1 | 1 | | 2 | 2 | | | 1 | | 1 | | | 1 | |
| Total score | 29 | | | | | | | | | | 27 | | | | | | | | | |
